# Respiratory syncytial virus disrupts the airway epithelial barrier by decreasing cortactin and destabilizing F-actin

**DOI:** 10.1242/jcs.259871

**Published:** 2022-08-16

**Authors:** Nannan Gao, Andjela Raduka, Fariba Rezaee

**Affiliations:** 1 Department of Inflammation and Immunity, Lerner Research Institute, Cleveland Clinic Foundation, Cleveland, Ohio 44195, USA; 2 Center for Pediatric Pulmonary Medicine, Cleveland Clinic Children's, Cleveland, Ohio 44195, USA

**Keywords:** Respiratory syncytial virus, RSV, Airway epithelial cells, Epithelial barrier, F-actin, Cortactin, Rap1 GTPase

## Abstract

Respiratory syncytial virus (RSV) infection is the leading cause of acute lower respiratory tract infection in young children worldwide. Our group recently revealed that RSV infection disrupts the airway epithelial barrier *in vitro* and *in vivo*. However, the underlying molecular pathways were still elusive. Here, we report the critical roles of the filamentous actin (F-actin) network and actin-binding protein cortactin in RSV infection. We found that RSV infection causes F-actin depolymerization in 16HBE cells, and that stabilizing the F-actin network in infected cells reverses the epithelial barrier disruption. RSV infection also leads to significantly decreased cortactin *in vitro* and *in vivo*. Cortactin-knockout 16HBE cells presented barrier dysfunction, whereas overexpression of cortactin protected the epithelial barrier against RSV. The activity of Rap1 (which has Rap1A and Rap1B forms), one downstream target of cortactin, declined after RSV infection as well as in cortactin-knockout cells. Moreover, activating Rap1 attenuated RSV-induced epithelial barrier disruption. Our study proposes a key mechanism in which RSV disrupts the airway epithelial barrier via attenuating cortactin expression and destabilizing the F-actin network. The identified pathways will provide new targets for therapeutic intervention toward RSV-related disease.

This article has an associated First Person interview with the first author of the paper.

## Abstract (precis)

**Summary:** A cortactin-dependent mechanism mediates disruption and dysfunction of epithelial barrier caused by respiratory syncytial virus infection.

## INTRODUCTION

The airway epithelium is the first line of defense in the respiratory system, and the integrity of the epithelial barrier is central to its functions in the innate immune responses and adaptive immune responses (Schleimer et al., 2007; Ganesan et al., 2013; Rezaee and Georas, 2014). Airway epithelial cells are major targets of inhaled environmental insults, such as respiratory viruses, and emerging evidence indicates that the breach of the epithelial barrier is a critical step during viral infection (Linfield et al., 2021b; Rezaee and Georas, 2014). One substantial contributing factor to the integrity and function of airway epithelium is the apical junctional complexes (AJCs) (Anderson et al., 2004; Rusu and Georgiou, 2020). The AJCs are located on the apical lateral side between adjacent epithelial cells, and it is composed of tight junctions (TJs) and adherens junctions (AJs). TJs and AJs establish cell–cell contact via intercellular homotypic and heterotypic interactions. TJs maintain the cell polarity by separating the apical and basolateral compartments of the membrane, and their tightness controls the transepithelial electrical resistance and the paracellular permeability, whereas AJs play an important role in regulating cell–cell adhesion (Niessen, 2007). Several transmembrane protein families are present in TJs, including members of the claudin family, the tight junction-associated marvel protein family, such as occludin, and the junctional adhesion molecules and coxsackie adenovirus receptor. Inside the cell, TJs are connected to the cytoplasmic scaffolding proteins, such as zonula occludens (ZO-1, ZO-2 and ZO-3; also known as TJP1–TJP3) and hence attached actin cytoskeleton. AJs contain members of two primary families, the cadherin family (such as E-cadherin) and the nectin family. Intracellularly, AJs bind to a cytoplasmic complex consisting of p120 catenin, β-catenin and α-catenin proteins, and are thus anchored to the actin cytoskeleton (Yuksel and Turkeli, 2017; Rusu and Georgiou, 2020; Rezaee and Georas, 2014; Linfield et al., 2021b). Emerging evidence indicates a critical role of AJCs in respiratory viral infection and reveals disassembled AJCs and increased barrier permeability as common characteristics for cells infected by respiratory viruses (Sajjan et al., 2008; Smallcombe et al., 2019; Rezaee et al., 2011; Singh et al., 2007; Short et al., 2016; Pharo et al., 2020; Teoh et al., 2010; De Maio et al., 2020; Chiu et al., 2014).

Respiratory syncytial virus (RSV) has been recognized as the leading cause of acute lower respiratory tract infection in young children worldwide (Hall et al., 2009; Geoghegan et al., 2017; Rezaee et al., 2017b; Meissner, 2016). Although RSV causes outbreaks of respiratory diseases annually across different age groups, it disproportionally affects infants, young children, older adults and immunocompromised individuals (Falsey et al., 2005; Hall, 2001, 2009). RSV infection is the major cause of hospitalization during infancy (Hall et al., 2013), and patients who suffer severe infections can be left with persistent wheezing or inflammatory airway diseases later in life (Carbonell-Estrany et al., 2015; Sigurs et al., 2010; Wu and Hartert, 2011). There is presently no vaccine or effective antiviral drug available for RSV (Rezaee et al., 2017b), underscoring the urgency and significance of understanding the pathological mechanism of RSV infection. RSV primarily infects epithelial cells in the airway, leads to distinct changes in epithelial cell morphology and physiology, and results in increased permeability of the epithelial barrier and widespread inflammation (Guo-Parke et al., 2013; Singh et al., 2007; Smallcombe et al., 2019). Previous studies have revealed that RSV infection increased the permeability of the airway epithelial barrier and disrupted the AJCs between epithelial cells *in vitro* (Rezaee et al., 2013; Singh et al., 2007; Rezaee et al., 2017a) and *in vivo* (Smallcombe et al., 2019). Despite the evident disassembly of AJCs during RSV infection, the underlying mechanism remains poorly understood.

The connection between TJs and AJs and the actin cytoskeleton is crucial to the structure and dynamic properties of the AJC, cellular polarity and tissue barrier (Hartsock and Nelson, 2008; Miyoshi and Takai, 2008; Rusu and Georgiou, 2020). It has been shown that RSV infection causes actin cytoskeleton reorganization (Linfield et al., 2021a; Rezaee et al., 2013), suggesting that the actin network is involved in AJC dysfunction after RSV infection. The cytoskeletal actin network is a dynamic structure that undergoes polymerization and depolymerization, and the balance is regulated by a variety of actin-binding proteins (Lee and Dominguez, 2010; Davidson and Wood, 2016). Among different actin-binding proteins, cortactin is a central player in organizing actin network structure and promoting actin assembly (Schnoor et al., 2018; Cosen-Binker and Kapus, 2006; Bougnères et al., 2004; Weed and Parsons, 2001). Studies have shown that cortactin-knockout (cortactin KO or KO) mice display increased vascular permeability (Schnoor et al., 2011), and cortactin is also necessary for intestinal epithelial barrier integrity *in vivo* and *in vitro* (Citalán-Madrid et al., 2017), suggesting that cortactin plays a critical role in regulating cell–cell adhesion. Previous studies have reported that cortactin maintains the activity of Rap1 (which has Rap1A and Rap1B forms) (Schnoor et al., 2011), a member of the Ras small GTPase family, which could promote actin polymerization together with another small GTPase Rac1 (van Buul and Timmerman, 2016; Caron, 2003). When activated by a cAMP analog, the Rap1/Rac1 pathway stabilizes the vascular endothelial barrier by enhancing actin polymerization (Adamson et al., 2008; Fukuhara et al., 2005).

The object of this study is to investigate the cellular and molecular mechanisms mediating AJC disassembly in airway epithelial cells during RSV infection. In this study, we examined the role of the actin cytoskeleton in RSV-mediated dysfunction of the airway epithelial barrier and disruption of AJC. We also investigated the effects of RSV infection on cortactin *in vitro* and *in vivo*. Utilizing cortactin overexpression and a cortactin-deficient airway epithelial cell line, we evaluated the function of cortactin in terms of AJC structure and barrier function. Finally, we elucidated that diminished Rap1 signaling is a downstream mechanism for the impacts of decreased cortactin during RSV infection. Our study concludes that RSV infection causes epithelial barrier deficits through attenuating cortactin/Rap1 signaling and destabilizing the actin network.

## RESULTS

### RSV infection depolymerizes F-actin in airway epithelial cells

**Fig. 1.**
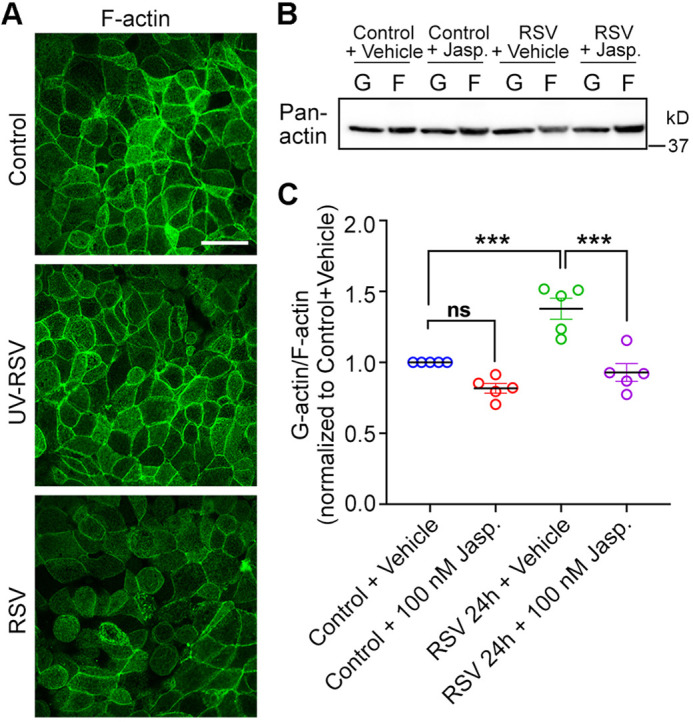
**RSV infection disrupts the F-actin network in airway epithelial cells.** (A) Confluent 16HBE cells were infected with control medium (control), UV-RSV or RSV at an MOI of 0.5. After 24 h of infection, cells were fixed with 4% PFA, F-actin network was probed by Alexa Fluor™ 633-phalloidin and visualized by confocal microscopy (visualized as green). Images are representative of three experiments. Scale bar: 25 µm. (B) Confluent 16HBE cells were infected with control medium or RSV for 22 h, then DMSO (vehicle) or 100 nM Jasp. was added for 2 h. Free globular-actin (G-actin, G) and filamentous actin (F-actin, F) fractions were separated from whole-cell lysates and blotted with anti-pan-actin antibodies. (C) The ratio of G-actin to F-actin from experiments shown in B was quantified using densitometry analysis and plotted as normalized values versus control+vehicle. Data shown as mean±s.e.m., *n*=5 independent experiments, repeated measures one-way ANOVA followed by Tukey's multiple comparisons test. Control+vehicle versus control+100 nM Jasp., not significant (ns), *P=*0.1038; control+vehicle versus RSV 24 h+vehicle, ***P<0.001 (*P=*0.0010); RSV 24 h+vehicle versus RSV 24 h+100 nM Jasp., ***P<0.001 (*P=*0.0002).

To investigate the role of the actin cytoskeleton in the airway epithelial cells during RSV infection, we first examined the structural changes of the actin network in RSV-infected 16HBE14o- (16HBE) cells (an immortalized human bronchial epithelial cell line). This cell line is a widely used *in vitro* model to study respiratory epithelial disease and barrier function (Callaghan et al., 2020; Smallcombe et al., 2020; Linfield et al., 2021a; Rezaee et al., 2017a). 16HBE cells were cultured on semipermeable membrane filters until they were confluent and formed a tight barrier with a transepithelial electrical resistance above 500 Ω cm^2^. Next, we inoculated cells with RSV [A2 strain, at a multiplicity of infection (MOI) of 0.5], ultraviolet-inactivated RSV (UV-RSV) or control medium (Control). This MOI was selected based on our previous studies (Rezaee et al., 2013, 2017a; Smallcombe et al., 2020) and was efficient in infecting airway epithelial cells without affecting cell proliferation or viability (Linfield et al., 2021a). UV-RSV was used as a control for viral replication because it has been shown to be alive but incapable of replicating as a result of UV-induced cross-linking (Jaovisidha et al., 1999). At 24 h after the inoculation, we examined the structure of the actin network by labeling cells with Alexa Fluor™-conjugated phalloidin (Fig. 1A), a highly selective probe toward actin filaments (F-actin). We found that RSV-infected cells demonstrated a marked decrease in F-actin staining compared with non-infected and UV-RSV-infected cells. The cellular actin cytoskeleton is consistently going through dynamic polymerization and depolymerization, and cells with enhanced actin depolymerization will exhibit reduced filamentous structure. Therefore, our data suggest that RSV infection depolymerizes F-actin in cultured airway epithelial cells. We also observed that F-actin morphology was similar between UV-RSV-infected cells and control cells, indicating the impact of RSV on the cellular actin network was dependent on active RSV replication. Furthermore, RSV-induced F-actin depolymerization was tested by measuring the change in the G-actin:F-actin ratio (Fig. 1B,C), a well-established approach of quantifying the F-actin turnover rate in cells (Gerits et al., 2007; Tu et al., 2003). Enhanced depolymerization of F-actin will result in more G-actin and less F-actin, leading to an increase in the G-actin:F-actin ratio; by contrast, increased polymerization or stabilization of F-actin will cause a decrease in the G-actin:F-actin ratio. We infected confluent 16HBE cells with control medium or RSV for 24 h and separated G-actin and F-actin fractions, and equal portions of both fractions were subjected to western blotting (Fig. 1B). The G-actin:F-actin ratio was then calculated by densitometric quantification of pan-actin (Fig. 1C). Results showed that the G-actin:F-actin ratio increased in RSV-infected cells compared with control cells, implying that RSV infection led to F-actin depolymerization in airway epithelial cells.

### F-actin depolymerization plays an important role in mediating airway epithelial barrier dysfunction caused by RSV

**Fig. 2.**
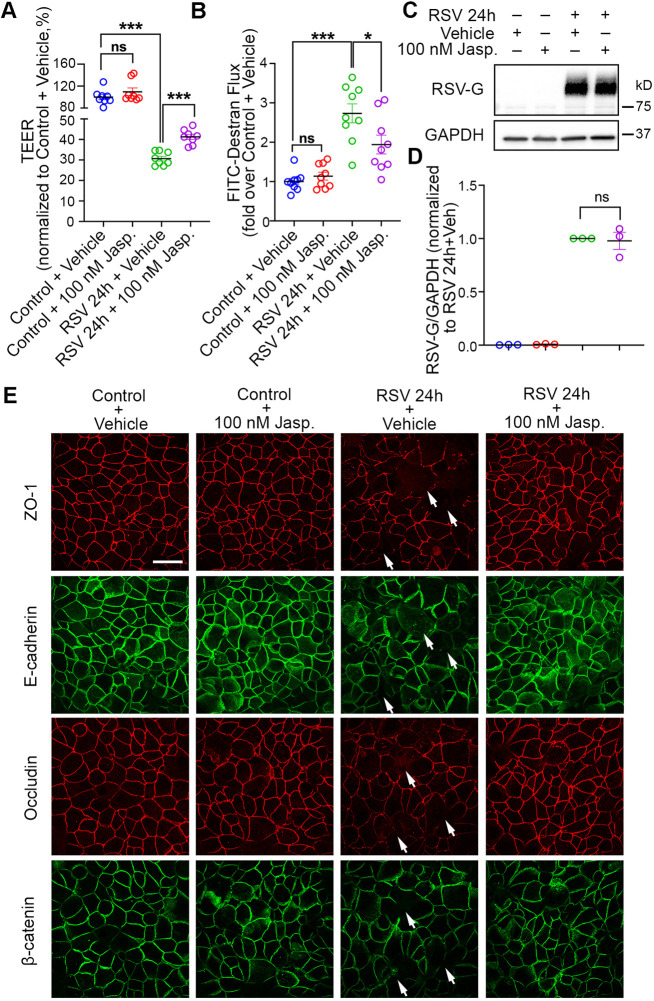
**Jasplakinolide treatment restores RSV-induced epithelial barrier disruption.** 16HBE cells were infected with RSV or control medium for 22 h, then DMSO (vehicle) or 100 nM Jasp. was added for another 2 h. (A) TEER (Ω cm^2^) was measured with the volt-ohm meter and plotted as percentage versus control+vehicle. Data shown as mean±s.e.m., *n*=8 replicates, one-way ANOVA followed by Tukey's multiple comparisons test. Control+vehicle versus control+100 nM Jasp., not significant (ns), *P=*0.6059; control+vehicle versus RSV 24 h+vehicle, ***P<0.001; RSV 24 h+vehicle versus RSV 24 h+100 nM Jasp., ***P<0.001. (B) The permeability of the epithelial barrier was quantified by measuring the transepithelial flux of 4 kDa FITC-conjugated dextran. Data are presented as normalized values versus control+vehicle. Note that the decreased resistance and increased permeability caused by RSV infection are attenuated in Jasp.-treated cells. Data shown as mean±s.e.m., *n*=9 replicates, one-way ANOVA followed by Tukey's multiple comparisons test. Control+vehicle versus control+100 nM Jasp., ns, *P=*0.9491; control+vehicle versus RSV 24 h+vehicle, ***P<0.001; RSV 24 h+vehicle versus RSV 24 h+100 nM Jasp., *P<0.05 (*P=*0.0207). (C,D) Jasplakinolide treatment does not affect RSV infection of 16HBE cells. (C) Whole-cell lysates were collected and subjected to western blotting with the indicated antibodies. GAPDH was used as the control for protein loading. (D) The expression of RSV-G glycoprotein was analyzed by densitometry and plotted as normalized values as indicated. Data shown as mean±s.e.m., *n*=3 independent experiments, paired two-tailed Student's *t*-test, not significant (ns), *P*=0.8105. (E) The structure of the AJC was determined by immunostaining with antibodies towards tight junction protein ZO-1 and occludin (red) as well as adherens junction protein E-cadherin and β-catenin (green). Arrows indicate disrupted AJCs in RSV-infected cells. Images are representative of four experiments. Scale bar: 25 µm.

To further understand the role of this cytoskeletal change in RSV infection, we turned to a pharmacological approach to stabilize actin filaments and examine the effect of modifying RSV-induced barrier dysfunction. Treatment with Jasplakinolide (Jasp., 100 nM), a commonly used actin stabilizer (Bubb et al., 1994; Visegrády et al., 2005; Posey and Bierer, 1999; Zhang et al., 2012), reduced the G-actin:F-actin ratio in RSV-infected cells (Fig. 1C, RSV+Jasp. versus RSV+vehicle), pointing to less F-actin depolymerization and validating that Jasp. could re-stabilize F-actin in RSV-infected cells. Next, we evaluated the function of the epithelial barrier by determining its electrical resistance and paracellular permeability (Fig. 2). Resistance was evaluated by measuring transepithelial electrical resistance (TEER), and paracellular permeability was determined by evaluating the paracellular flux of FITC–dextran (4 kDa). Intact TJs and a less ‘leaky’ barrier will exhibit a higher TEER and lower flux of FITC–dextran. RSV-infected cells exhibited decreased TEER (Fig. 2A) and increased FITC–dextran flux (Fig. 2B), indicating a significantly disrupted barrier integrity. Re-stabilizing F-actin with Jasp. treatment resulted in a higher TEER (Fig. 2A) and lower dextran flux (Fig. 2B) in RSV-infected cells, implying a less ‘leaky’ barrier when F-actin was stabilized during RSV infection. Moreover, Jasp. treatment did not impact the level of RSV-glycoprotein G (RSV-G) in RSV-infected cells (Fig. 2C,D), indicating that the preventive effect of Jasp. in RSV-mediated barrier dysfunction was not due to reduced RSV infection. When the AJC structure was examined by immunolabeling of the TJ proteins ZO-1 and occludin, as well as AJ proteins E-cadherin and β-catenin, control cells exhibited a uniform ‘chicken wire’ pattern of these TJ and AJ proteins (Fig. 2E), which was significantly disrupted after RSV infection for 24 h. F-actin stabilization by means of Jasp. restored these disrupted patterns, indicating improved AJC structure when stabilizing F-actin during RSV infection. Taken together, these data reveal that F-actin depolymerization is a critical downstream mechanism for RSV-mediated epithelial barrier permeability and AJC disassembly.

### RSV infection decreases cortactin in airway epithelial cells *in vitro* and *in vivo*

**Fig. 3.**
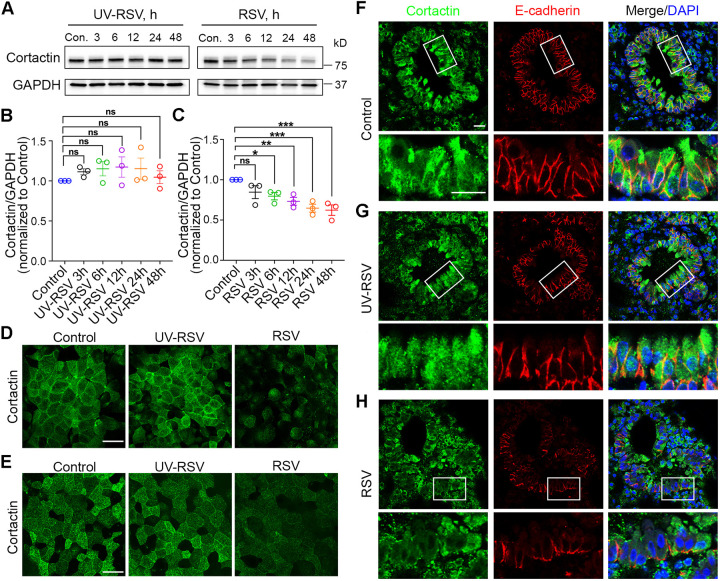
**Cortactin decreases during RSV infection in airway epithelial cells *in vitro* and *in vivo*.** (A) Representative western blot of whole-cell lysates from 16HBE cells infected with control medium (Con.), UV-RSV or RSV for 0–48 h. GAPDH served as the control for protein loading. (B,C) Densitometry analysis of blots from experiments as in A. The protein level of cortactin was determined by densitometric analysis and plotted as normalized values as indicated. Data shown as mean±s.e.m., *n*=3 independent experiments, repeated measures one-way ANOVA followed by Dunnett's multiple comparisons test. Control versus UV-RSV 3 h, not significant (ns), *P*=0.7255; control versus UV-RSV 6 h, ns, *P*=0.5035; control versus UV-RSV 12 h, ns, *P*=0.3928; control versus UV-RSV 24 h, ns, *P*=0.4937; control versus UV-RSV 48 h, ns, *P*=0.9883. Control versus RSV 3 h, ns, *P*=0.1151; control versus RSV 6 h, *P<0.05 (*P*=0.0319); control versus RSV 12 h, **P<0.01 (*P*=0.0062); control versus RSV 24 h, ***P<0.001 (*P*=0.0009); control versus RSV 48 h, ***P<0.001 (*P*=0.0005). (D) 16HBE cells were infected with control medium (Con.), UV-RSV or RSV for 24 h and fixed, cells were immunolabeled with antibodies against cortactin and imaged by confocal microscopy (green). (E) Primary NHBE cells were differentiated at the air–liquid interface for 33 days and infected with control medium, UV-RSV or RSV (MOI=2). At 4 days after infection, cells were fixed, immunostained with antibodies against cortactin, and imaged by confocal microscopy (green). (F–H) Representative images of lung tissue from mice inoculated with control (RSV growing medium) (F), UV-RSV (G), or RSV (H). Mice were intranasally inoculated and euthanized 4 days later. Lung tissues were harvested and sections were subjected to immunohistochemistry using antibodies again cortactin (green) and E-cadherin (red). Enlarged images of small airways from the indicated area are shown underneath overview images. Images in D–H are representative of at least three experiments. Scale bars: 25 µm.

Previous studies have shown that loss of cortactin leads to disruption of the vascular endothelial barrier (Schnoor et al., 2011) and intestinal epithelial barrier (Citalán-Madrid et al., 2017). To investigate the underlying mechanism for RSV-induced F-actin d­epolymerization, we examined whether there was any change in cortactin. Western blot data showed that, in 16HBE cells, the protein level of cortactin evidently decreased after RSV infection in a time-dependent manner (Fig. 3A–C), suggesting cortactin might be involved in cellular changes following RSV infection. Moreover, we accessed the expression of cortactin through immunostaining in both *in vitro* and *in vivo* models for RSV infection (Fig. 3D–H). In RSV-infected 16HBE cells, we noticed that the immunofluorescent intensity of cortactin was significantly reduced compared to that in control and UV-RSV-infected cells (Fig. 3D). Similarly, in primary pediatric human epithelial cells (normal human bronchial epithelial, NHBE) cultured at an air–liquid interface, we observed that cortactin expression decreased in RSV-infected cells versus control and UV-RSV infected cells (Fig. 3E). Likewise, in a recently established rodent model for RSV infection (Smallcombe et al., 2019), immunohistochemistry staining showed decreased cortactin in small airways from RSV-infected lungs, especially in the epithelial cells (shown as E-cadherin-positive) (Fig. 3F,H). UV-RSV infection did not result in reduced cortactin *in vitro* nor *in vivo* (Fig. 3D,E,G), suggesting that viral replication was necessary for this effect, similar to what we found about RSV's effect on actin depolymerization (Fig. 1). Collectively, these results suggest an important part played by cortactin in the airway epithelium following RSV infection.

### Deficiency in cortactin increases the permeability of the airway epithelial barrier

**Fig. 4.**
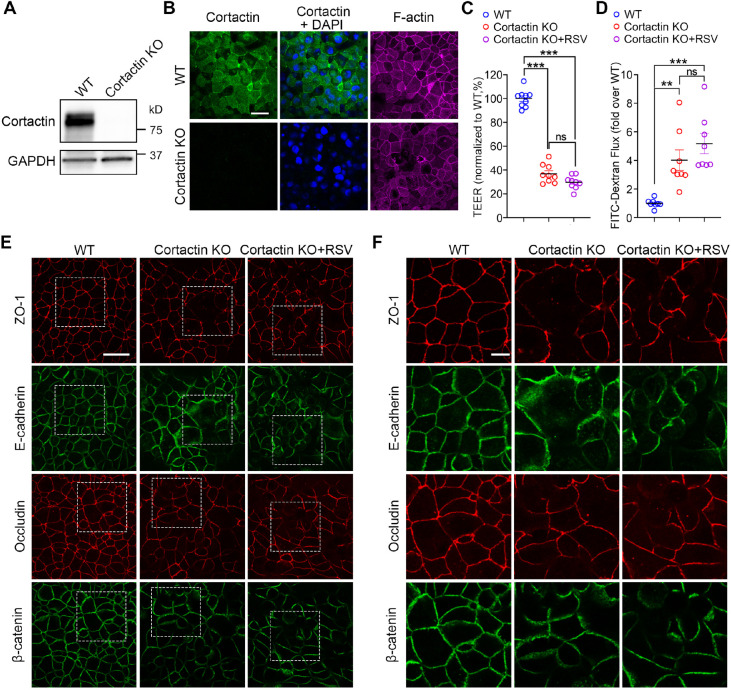
**Epithelial barrier integrity is disrupted in cortactin KO 16HBE cells.** (A) Representative western blot of whole-cell lysates from WT and cortactin KO 16HBE cells. GAPDH served as the control for protein loading. Image representative of four experiments. (B) WT and cortactin KO 16HBE cells were fixed with 4% PFA, labeled with antibodies or probes, and imaged by confocal microscopy. Cortactin was immunolabeled with antibodies and visualized as green, nuclei were labeled by DAPI and visualized as blue; the F-actin network was probed by Alexa Fluor™ 633-phalloidin and visualized as magenta. Images are representative of three experiments. Scale bar: 25 µm. (C,D) 16HBE cells of the indicated genotype were seeded at the same density and cultured until they were confluent; RSV-infected cortactin KO cells were infected by RSV for 24 h. Cortactin KO epithelial cells presented disrupted barrier integrity, shown as reduced resistance and increased permeability compared with WT cells, and RSV did not cause significant additional impacts on the barrier function in cortactin KO cells. (C) TEER (Ω cm^2^) was measured by a volt-ohm meter and plotted as a percentage versus WT. Data shown as mean±s.e.m., *n*=9 replicates from three independent KO clones, one-way ANOVA followed by Tukey's multiple comparisons. WT versus cortactin KO, ***P<0.001; WT versus cortactin KO+RSV, ***P<0.001; cortactin KO versus cortactin KO+RSV not significant (ns), *P*=0.0948. (D) The permeability of the epithelial barrier was evaluated by the transepithelial flux of 4 kDa FITC-conjugated dextran and normalized to WT. Data shown as mean±s.e.m., *n*=8 replicates from three independent KO clones, one-way ANOVA followed by Tukey's multiple comparisons. WT versus cortactin KO, **P<0.01 (*P=*0.0074); WT versus cortactin KO+RSV, ***P<0.001 (*P=*0.0007); cortactin KO versus cortactin KO+RSV not significant (ns), *P*=0.2198. (E,F) The structure of the AJC was determined by immunostaining with antibodies towards tight junction protein ZO-1 and occludin (red) as well as the AJ junction proteins E-cadherin and β-catenin (green). Higher magnifications of the areas indicated are E are shown in F. Scale bars: 25 μm (E), 10 µm (F).

RSV infection has been shown to cause airway epithelial barrier dysfunction and structural disruption (Smallcombe et al., 2019; Rezaee et al., 2013, 2017a). Considering the significant decline in cortactin both *in vitro* and *in vivo* after RSV infection (Fig. 3), we reason that cortactin is necessary for the integrity of the airway epithelial barrier and that the decrease of cortactin is a downstream mechanism for RSV infection. To test this hypothesis, we generated cortactin KO 16HBE cells using the CRISPR-Cas9 system targeting exons 3 and 12 of the human *CTTN* gene (Fig. 4; Fig. S1). Successful deletion of cortactin from single 16HBE clones was validated by western blotting (Fig. 4A), genomic DNA sequence analysis (Fig. S1A), and immunostaining towards cortactin (Fig. 4B) in comparison with wild-type (WT) cells. To rule out non-specific off-target effects, data and representative images were collected from three different KO clones. We noticed decreased F-actin staining in cortactin KO cells (Fig. 4B), similar to our observations in RSV-infected cells (Fig. 1A). The TEER of cortactin KO cells significantly decreased compared to WT cells (Fig. 4C), and a marked increase in dextran efflux was also demonstrated by cortactin KO 16HBE cells (Fig. 4D). These deficits in barrier permeability indicated impaired barrier integrity in cortactin KO cells. Meanwhile, cortactin KO 16HBE monolayers were subjected to immunostaining of TJ and AJ proteins. As shown in Fig. 4E,F and Fig. S1C,D, the patterns of TJ proteins (ZO-1 and occludin) and AJ proteins (E-cadherin and β-catenin) in cortactin KO cells were disrupted when compared with WT cells, indicating disassembly of the AJC structure in the absence of cortactin. Altogether, these data suggest that cortactin is critical for barrier function in epithelial cells. Moreover, RSV infection of cortactin KO cells was comparable to that of WT cells (Fig. S1B), and the impairments in barrier integrity displayed by cortactin KO cells did not exhibit further deterioration after RSV infection (Fig. 4C–F and Fig. S1C,D, cortactin KO versus cortactin KO+RSV), supporting the notion that deficiency in cortactin is a critical downstream mechanism for RSV infection.

### Increasing cortactin is protective of the airway epithelial barrier against RSV

**Fig. 5.**
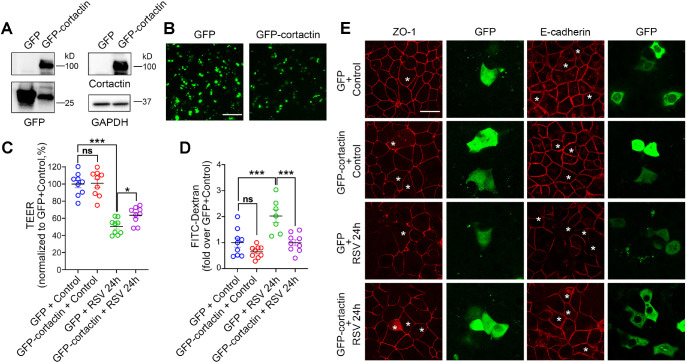
**Cortactin overexpression attenuates RSV-induced epithelial barrier dysfunction.** Non-confluent 16HBE cells were transfected with GFP plasmid or GFP–cortactin plasmid using Lipofectamine^TM^ 3000 reagent. Cells were infected with RSV or control medium 40 h after the transfection. (A) Representative western blot of whole-cell lysates from 16HBE cells transfected with GFP or GFP–cortactin plasmids. GAPDH served as the control for protein loading. Images are representative of two experiments. (B) The expression of GFP or GFP-cortactin in transfected 16HBE cells was directly visualized by imaging the Transwell inserts with an inverted fluorescence microscope without staining (green). Images are representative of four experiments. Scale bar: 50 µm. (C) TEER (Ω cm^2^) was measured by a volt-ohm meter and plotted as percentage versus GFP+control. Data shown as mean±s.e.m., *n*=9 replicates, one-way ANOVA followed by Tukey's multiple comparisons test. GFP+control versus GFP-cortactin+control, not significant (ns), *P*=0.9880; GFP+control versus GFP+RSV 24 h, ***P<0.001; GFP+RSV 24 h versus GFP–cortactin+RSV 24 h, *P<0.05 (*P*=0.0122). (D) Permeability of the epithelial barrier was quantified by the transepithelial flux of 4 kDa FITC-conjugated dextran and normalized to GFP+control. Data shown as mean±s.e.m., *n*=7 or 9 replicates, one-way ANOVA followed by Tukey's multiple comparisons test, GFP+control versus GFP–cortactin+control, not significant (ns), *P*=0.3566; GFP+control versus GFP+RSV 24 h, ***P<0.001 (*P*=0.0005); GFP+RSV 24 h versus GFP–cortactin+RSV 24 h, ***P<0.001 (*P*=0.0005). (E) The structure of the AJC was determined by immunostaining with antibodies towards ZO-1 or E-cadherin (red) along with GFP (green). Asterisks indicate cells transfected by indicated plasmids. Images are representative of three experiments. Scale bar: 25 µm.

To further address whether reduced cortactin is a downstream mechanism for RSV infection, we increased the level of cortactin by means of overexpression plasmids encoding GFP-fused human cortactin (GFP–cortactin) in 16HBE cells (Fig. 5). Cells were cultured on Transwell inserts and transfected with GFP or GFP–cortactin plasmids, then cells were cultured until 100% confluency and inoculated with RSV or control medium for 24 h. The expressions of both plasmids were examined by detecting GFP and cortactin proteins at the correct molecular mass (GFP, ∼25 kDa, human cortactin, ∼80 kDa, Fig. 5A), as well as the GFP fluorescence signals on Transwell inserts (Fig. 5B). In the lysates of GFP–cortactin cells, we noticed bands at lower molecular masses when blotting with anti-GFP antibodies (Fig. 5A; Fig. S2E), which could be due to the degradation of cortactin caused by RSV infection. We found that overexpression of cortactin mitigated the decreased TEER in RSV-infected cells (Fig. 5C). The increase in dextran efflux caused by RSV infection was also alleviated in cortactin overexpressing cells (Fig. 5D). These observations suggest a protective effect of increasing cortactin on RSV-induced barrier permeability. We also evaluated the structural integrity of the epithelial barrier through immunostaining against TJ and AJ proteins. As shown in Fig. 5E, overexpression of cortactin also prevented the disrupted patterns of ZO-1 and E-cadherin in RSV-infected cells in the areas containing the GFP-cortactin-positive cells (indicated by asterisks) to some extent. Altogether, these results suggest that increasing cortactin protects the airway epithelial barrier against RSV infection, implying that cortactin acts downstream of the RSV infection in the airway epithelial cells.

### RSV induces epithelial barrier disruption via diminishing Rap1 activity

So far, our data has revealed the critical roles of the F-actin network and cortactin in the airway epithelial barrier during RSV infection. Given that the cortactin/Rap1 pathway maintains barrier integrity in vascular endothelial cells (Schnoor et al., 2011) and Rap1 promotes actin polymerization (Ivanov et al., 2005; van Buul and Timmerman, 2016; Caron, 2003), we reasoned that RSV disturbs the airway epithelial barrier via attenuating the cortactin/Rap1/Rac1 pathway.

**Fig. 6.**
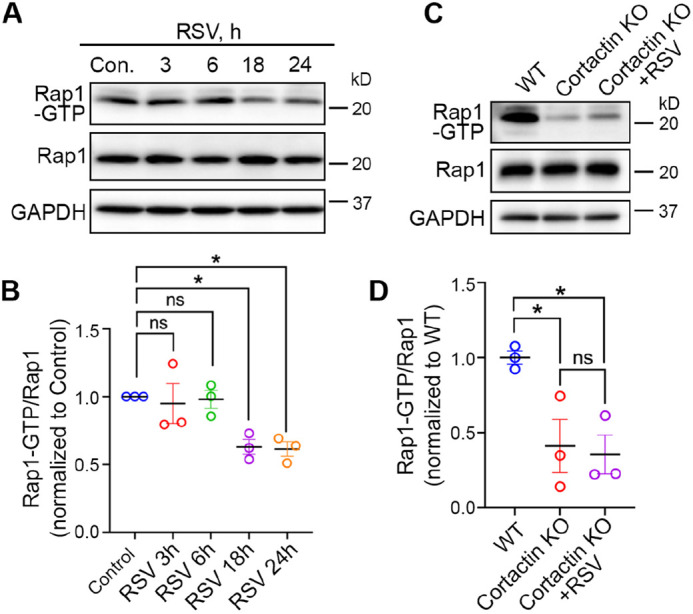
**Rap1 activity decreases during RSV infection and in the absence of cortactin.** (A) 16HBE cells were infected with control medium (Con.) or RSV for the indicated period and subsequently subjected to Rap1 activity assays. Rap1-GTP was pulled down from cell lysates with GST-RalGDS RBD beads and analyzed by immunoblotting with anti-Rap1 antibodies. Rap1 was analyzed in the whole-cell lysate. GAPDH was used as the control for protein loading. (B) Quantification of immunoblots from experiments as in A. The ratio of Rap1-GTP to Rap1 was determined by densitometric analysis and plotted as normalized values versus control. Data shown as mean±s.e.m., *n*=3 independent experiments, repeated measures one-way ANOVA followed by Dunnett's multiple comparisons. Control versus RSV 3 h, not significant (ns), *P*=0.9855; control versus RSV 6 h, ns, *P*=0.9919; control versus RSV 18 h, *P<0.05 (*P*=0.0475); control versus RSV 24 h, *P<0.05 (*P*=0.0417). (C) WT, cortactin KO and RSV-infected cortactin KO 16HBE cells were subjected to Rap1 activity assays. Rap1-GTP was pulled down from cell lysates with GST-RalGDS RBD beads and analyzed by immunoblotting with anti-Rap1 antibodies. Rap1 was analyzed in the whole-cell lysate. GAPDH was used as the control for protein loading. (D) Quantification of immunoblots from experiments as in C. The Rap1-GTP to Rap1 ratio was determined by densitometric analysis and normalized to WT. Data shown as mean±s.e.m., *n*=3 replicates from three independent KO clones, one-way ANOVA followed by Tukey's multiple comparisons. WT versus cortactin KO, *P<0.05 (*P*=0.0411); WT versus cortactin KO+RSV, *P<0.05 (*P*=0.0284); cortactin KO versus cortactin KO+RSV not significant (ns), *P*=0.9490.

To test our hypothesis, we examined whether the Rap1 signaling pathway was altered during RSV infection. Similar to other small GTPases in the Ras family, Rap1 functions as a molecular switch by transiting between two states – an inactive GDP-bound state and an active GTP-bound state (Jaśkiewicz et al., 2018). As the active form of Rap1, Rap1-GTP has been used as an indicator for Rap1 activity (Jeon et al., 2007; McLeod et al., 2004). We determined the level of Rap1-GTP from 16HBE cells that were infected for different periods by RSV (Fig. 6A). RalGDS Rap1-GTP-binding domain (RalGDS RBD) agarose beads were used to selectively pull down Rap1-GTP from cell lysates, and the precipitated Rap1-GTP was detected by western blotting using anti-Rap1 antibodies. Densitometric quantification showed that Rap1-GTP declined in cells infected by RSV for 18 h and 24 h compared with uninfected cells (Fig. 6B), indicating that RSV reduced Rap1 signaling activity in airway epithelial cells. Interestingly, we also found a reduced Rap1-GTP in cortactin KO cells similar to that induced by RSV infection (Fig. 6C,D, WT versus cortactin KO). Additionally, we assessed the activity of Rap1 in RSV-infected KO cells, and no significant additive decrease in Rap1 activity was observed compared to KO cells (Fig. 6C,D, cortactin KO versus cortactin KO+RSV). Collectively, these data suggest that cortactin is a major downstream factor mediating RSV-induced barrier disruption.

**Fig. 7.**
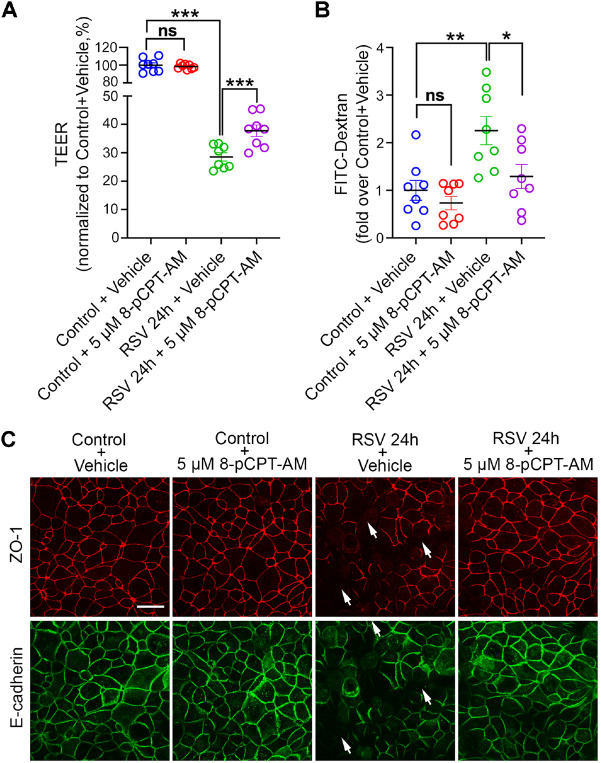
**Rap1 activation mitigates the epithelial barrier dysfunction caused by RSV infection.** 16 HBE cells were infected with RSV or control medium for 22 h and DMSO (vehicle) or 5 µM 8-pCPT-AM was added for another 2 h. Treatment of 8-pCPT-AM alleviated reduced resistance and increased permeability caused by RSV. (A) TEER (Ω cm^2^) was measured with a volt-ohm meter and plotted as percentage versus control+vehicle. Data shown as mean±s.e.m., *n*=8 replicates, one-way ANOVA followed by Tukey's multiple comparisons test. Control+vehicle versus control+5 µM 8-pCPT-AM, not significant (ns), *P*=0.9184; control+vehicle versus RSV 24 h+vehicle, ***P<0.001; RSV 24 h+vehicle versus RSV 24 h+5 µM 8-pCPT-AM, ***P<0.001 (*P*=0.0007). (B) Permeability of the epithelial barrier was quantified by measuring the transepithelial flux of 4 kDa FITC-conjugated dextran and normalized to control+vehicle. Data shown as mean±s.e.m., *n*=8 replicates, one-way ANOVA followed by Tukey's multiple comparisons test. Control+vehicle versus control+5 µM 8-pCPT-AM, not significant (ns), *P*=0.8495; control+vehicle versus RSV 24 h+vehicle, **P<0.01 (*P*=0.0036); RSV 24 h+vehicle versus RSV 24 h+5 µM 8-pCPT-AM, *P=0.0321. (C) The structure of the AJC was determined by immunostaining with antibodies towards tight junction protein ZO-1 (red) and adherens junction protein E-cadherin (green). Arrows indicate disrupted AJCs in RSV-infected cells. Images are representative of three experiments. Scale bar: 25 µm.

To further examine the necessary role of the Rap1 signaling pathway in the breakdown and dysfunction of the epithelial barrier, we treated RSV-infected cells with the Rap1 activator 8-pCPT-2-O-Me-cAMP-AM (8-pCPT-AM) (Fig. 7). As a selective cell-permeable activator for Epac (also known as RAPGEF3), 8-pCPT-AM induces robust Rap1 activation when administrated *in vitro* (Chepurny et al., 2009; Vliem et al., 2008). Compared with vehicle (DMSO), 5 µM 8-pCPT-AM partially restored the epithelial barrier integrity, as evidenced by the improved TEER (Fig. 7A) and alleviated efflux of dextran (Fig. 7B) in RSV-infected cells after treatment. Consistent with this, immunostaining towards ZO-1 and E-cadherin revealed that RSV-induced disruption in the AJC structure was also attenuated by 8-pCPT-AM treatment (Fig. 7C). Altogether, these results indicate that the reduction in Rap1 activity caused by RSV infection is critical for the RSV-induced disassembly of the AJC and dysfunction of the epithelial barrier.

## DISCUSSION

**Fig. 8.**
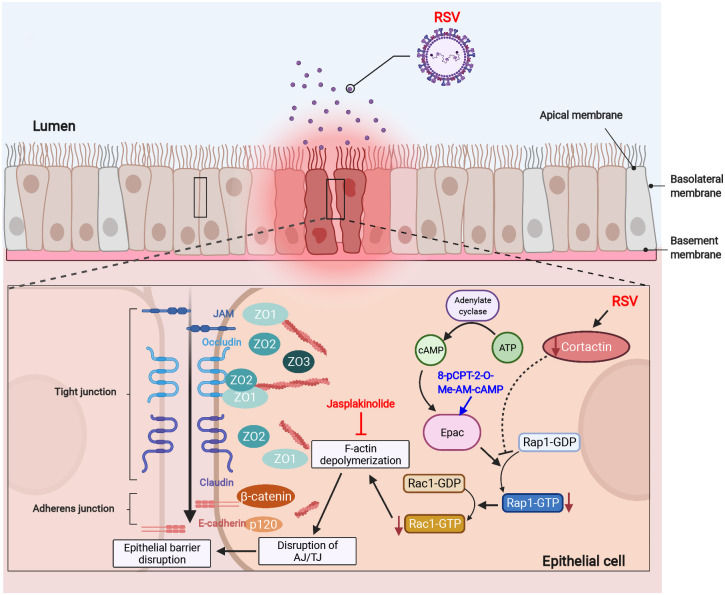
**A graphical model showing mechanism through which RSV infection induces epithelial barrier dysfunction.** RSV infection disrupts the epithelial barrier via attenuating the cortactin/Rap1 signaling pathway and depolymerizing F-actin. In RSV-infected epithelial cells, the protein level of cortactin reduces, which in turn attenuates the activity of the Rap1/Rac1 signaling pathway and leads to F-actin depolymerization. Destabilization of the actin network finally results in disrupted structure and increased permeability of the airway epithelial barrier through disassembly of the AJC, which is extensively associated with the F-actin network. These RSV-induced barrier dysfunctions will be mitigated via activation of Rap1 signaling by Epac activator 8-pCPT-2-O-Me-cAMP-AM or F-actin stabilization by jasplakinolide. This image was created with BioRender.com.

Tightly packed epithelial cells form a barrier on the surface of the airway that is exposed to the outside world, protecting us against inhaled insults such as respiratory viruses. Emerging evidence from our laboratory and others has demonstrated that the increased permeability of the airway epithelial barrier and disassembly of the AJC is among the most evident impacts of RSV infection (Linfield et al., 2021b; Rezaee and Georas, 2014). However, the molecular and cellular mechanisms underlying these deficits are far from fully understood. Here, we report that RSV induces epithelial barrier dysfunction via a cortactin-dependent mechanism. In the airway epithelial cells, RSV infection decreases cortactin, thus inhibiting cAMP/Rap1/Rac1 signaling and leading to F-actin depolymerization. The destabilized F-actin network ultimately induces disassembly of the AJC and the increased permeability of the epithelial barrier (Fig. 8).

The key to the function of the airway epithelial barrier is a specialized intercellular junction referred to as the AJC, which is located on the apical lateral side of the epithelial cells (Niessen, 2007). Through protein–protein interactions, transmembrane components and scaffolding proteins of the AJC establish the cell–cell connection (Ganesan et al., 2013; Rezaee and Georas, 2014). Inside the cell, AJC is anchored to the cytoskeleton network, especially the perijunctional actin cytoskeleton (Zihni et al., 2016). Numerous pieces of evidence have suggested a critical role of the actin cytoskeleton in regulating TJ and AJ structure and function (Hartsock and Nelson, 2008; Rusu and Georgiou, 2020). It has been reported that anchoring to the actin cytoskeleton significantly increases the assembly and stability of the cadherin cluster and AJ (Hong et al., 2013). In the TJ, ZO proteins bind to F-actin directly and act as scaffolds for occludin and claudins to bind to the actin cytoskeleton (Fanning et al., 1998; Wittchen et al., 1999; Van Itallie et al., 2017). Moreover, the actin-binding domain of ZO-1 is required for its localization to TJ (Fanning et al., 2002). During viral infection, viruses engage cellular actin cytoskeleton and subvert the actin cytoskeleton in the host cell from entry to egress (Taylor et al., 2011). Studies have revealed that RSV utilizes F-actin to enter and release from the host cell (Shahriari et al., 2016; El Najjar et al., 2014; Kallewaard et al., 2005), as well as to transport RSV components to the budding site (Shahriari et al., 2018; Ghildyal et al., 2012). However, the mechanisms by which RSV-induced disruption of the cellular actin network leads to AJC disruption and airway barrier dysfunction are not well understood. In this study, our data show that RSV infection leads to depolymerization of the F-actin network in airway epithelial cells (Fig. 1). A similar decrease in F-actin has been described in differentiated primary normal human bronchial epithelial cells infected by the influenza H1N1 virus (Pharo et al., 2020). Additionally, disassembly of F-actin has been reported in differentiated primary human airway epithelial cells after measles virus infection (Singh et al., 2019). We show that pharmacologically stabilizing F-actin with Jasp. treatment restores epithelial barrier structure and function in RSV-infected 16HBE cells (Fig. 2), implying that F-actin depolymerization is a critical downstream mechanism for RSV-mediated epithelial barrier permeability and AJC disassembly.

The cellular actin network is a dynamic structure that is capable of remodeling quickly in response to different cellular processes and extracellular stimuli (Blanchoin et al., 2014). The dynamic behaviors and organization of the actin cytoskeleton play a key role in regulating the function of the epithelial barrier (as described above) and are tightly regulated by a variety of actin-binding proteins and signaling (Schmidt and Hall, 1998). Cortactin, one such regulator, was discovered as a filamentous actin-binding protein (Wu and Parsons, 1993; Wu et al., 1991) and has been implicated as a key player in a wide range of cellular processes involving actin cytoskeleton remodeling, such as host–pathogen interactions and the establishment of cell–cell contacts (Ammer and Weed, 2008; Schnoor et al., 2018; Cosen-Binker and Kapus, 2006). In particular, cortactin regulates the organization of the AJC at intercellular junctions. Cortactin has been found to associate with ZO-1 at the TJs (Katsube et al., 1998) and associated with E-cadherin in epithelial cells (Sroka et al., 2016) and N-cadherin in fibroblasts (El Sayegh et al., 2004) at the AJs. A previous study revealed the role of cortactin in mediating the increase in TEER evoked by sphingosine-1 phosphate in lung endothelial cells (Dudek et al., 2004). Knocking down cortactin or disrupting the binding of cortactin to F-actin in epithelial cells inhibited the E-cadherin adhesion and biogenesis of AJs between adjacent epithelial cells (Helwani et al., 2004). Moreover, RNAi-mediated depletion of cortactin in fibroblasts has shown that cortactin is critical for the N-cadherin-mediated intercellular adhesion strength (El Sayegh et al., 2004).

Despite the growing body of knowledge on the function of cortactin, relatively little is known regarding the involvement of cortactin in barrier dysfunction caused by viral infection. Our data identify that there is a decrease in the expression of cortactin during RSV infection both *in vitro* and *in vivo* (Fig. 3). Similarly, a decrease in cortactin was observed during persistent morbillivirus infection in histiocytic sarcoma cells (Pfankuche et al., 2016). The level of cortactin was also found to be downregulated in influenza A virus-infected MDCK and A549 cells 24 h after infection (Chen and Husain, 2016). We found that depletion of cortactin in 16HBE cells leads to AJC disassembly as well as barrier dysfunction (Fig. 4; Fig. S1). Congruously, previous studies have suggested that ablation of cortactin in mice increased the permeability of the vascular endothelial barrier (Schnoor et al., 2011) and intestinal epithelial barrier (Citalán-Madrid et al., 2017). Additionally, downregulation of cortactin caused barrier dysfunction in Caco-2 cells (Citalán-Madrid et al., 2017), whereas RSV infection did not cause additional disruption of the barrier function in cortactin KO cells (Fig. 4; Fig. S1). Additionally, increasing cortactin levels before RSV infection shows a protective effect on AJC structure and barrier permeability against the virus (Fig. 5). Collectively, our data suggest that cortactin is a critical downstream effector of RSV infection in airway epithelial cells. Although our study sheds light on the important role of actin-binding proteins in the epithelial response to viral infection, we acknowledge that the mechanistic link between RSV infection and cortactin decrease is still missing in the current study, and warrants further investigation. A recent study by Hunziker et al. elucidated a shift in cortactin phosphorylation patterns in response to Influenza A virus (IAV) infection within minutes (Hunziker et al., 2022). They found that IAV induced Cdc42-dependent filopodia formation that enhanced virus uptake in the airway epithelial cells, whereas cortactin translocated to the entry sites of IAV, prevented the formation of filopodia and thus restricted virus endocytosis through dynamic phosphorylation. Hence, the decline of cortactin observed in this study might also facilitate RSV entry. Although we recognize that dynamic phosphorylation could serve as a general mechanism among different viral infections, we have not investigated the changes in phosphorylation of the remaining cortactin after RSV infection, leaving it a potential subject for future studies. As for the absence of cortactin re-localization after viral infection reported by Hunziker et al. (2022), we did not focus on the first 10–20 min within RSV infection because the disruption of the barrier happens later. The change in cortactin relocation might be a time frame-sensitive phenomenon and therefore was not captured by our experiments.

Although cortactin was originally identified as a nucleation-promoting factor for actin, emerging evidence reveals its role in regulating cellular signaling pathways. For example, cortactin has been found necessary for the basal activity level of Rap1 in endothelial cells (García Ponce et al., 2016; Schnoor et al., 2011). Rap1 is a small GTPase belonging to the Ras family, which promotes actin polymerization (van Buul and Timmerman, 2016; Caron, 2003). These findings prompted us to examine the role of the Rap1 pathway in RSV-induced disturbance of the airway epithelial barrier. The decrease of cortactin in RSV-infected cells is accompanied by an attenuated Rap1 activity, and Rap1 activity also decreased in cortactin KO cells (Fig. 6). Rap1 activation by 8-pCPT-AM could mitigate the barrier dysfunction and AJC disruption caused by RSV infection (Fig. 7). In line with our findings, activating the Rap1/Rac1 pathway stabilized the vascular endothelial barrier by enhancing actin polymerization (Adamson et al., 2008; Fukuhara et al., 2005). How cortactin regulates Rap1 is an important question to pursue in future studies. Indeed, cortactin is a substrate for multiple serine/threonine kinases [e.g. ERKs, protein kinase D (PKD) and PAK] in addition to serving as a tyrosine kinase substrate (e.g. for Src, Fer and Syk), such signaling pathways might serve as the aforementioned mechanistic connections (Martinez-Quiles et al., 2004).

We noticed that the extents of improvement of TEER in RSV-infected cells by pharmacological treatments or cortactin overexpression were statistically significant, but limited as compared to the extents of decrease of FITC-dextran flux among several experiments (Figs 3, 5 and 7). These discrepancies could be due to the size differences between the molecules these two assays measured. Although they are both indicators of paracellular permeability, the electrical resistance is a measurement of the transjunctional flow of ions, whereas dextran permeability characterizes the flux of high molecular mass molecules across the cellular barrier (Bednarek, 2022). Therefore, the results from these assays are not linearly correlated.

In conclusion, our data reveal how a common respiratory virus destabilizes the F-actin network and therefore disrupts the structure of the AJC, which ultimately impairs the function of the airway epithelial barrier. Our findings unravel a novel mechanism in which RSV induces airway epithelial barrier dysfunction through attenuating cortactin expression and inhibiting Rap1 signaling. This study sheds light on how RSV results in a ‘leaky’ epithelial barrier, as well as on the previously unexamined role of cortactin in mediating the impact of RSV. The identified pathways will provide new targets for therapeutic intervention and the potential for positively impacting the management of RSV diseases.

## MATERIALS AND METHODS

### Antibodies, reagents and plasmids

Primary antibodies against the following proteins were used: cortactin (05-180, Millipore Sigma, Darmstadt, Germany; ab33333, Abcam, Cambridge, UK), glyceraldehyde 3-phosphate dehydrogenase (GAPDH) (ab8245, Abcam), pan-actin (A2103, Sigma-Aldrich, St Louis, MO), ZO-1 (33-9100 or 40-2200, Invitrogen, Waltham, MA), E-cadherin (ab40772, Abcam; 610181, BD Biosciences, Franklin Lakes, NJ; 3195S, Cell Signaling Technology, Danvers, MA), occludin (33-1500, Invitrogen), β-catenin (ab32572, Abcam), RSV G protein (A2) (GTX70381, GeneTex, Irvine, CA), GFP (ab290, Abcam), Rap1 (07-916, Millipore Sigma). Detailed information such as clone number, validation, and dilution was included in Table S1. The following secondary antibodies were obtained from Invitrogen: Alexa Fluor™ 488 anti-rabbit IgG (A21206), Alexa Fluor™ 488 anti-mouse IgG (A21202), Alexa Fluor™ 568 anti-rabbit IgG (A10042), and Alexa Fluor™ 568 anti-mouse IgG (A10037) antibodies, used for staining (1:500); horseradish peroxidase (HRP)-conjugated goat anti-rabbit IgG (31460) and HRP-conjugated goat anti-mouse IgG (31430) antibodies were used for western blotting (1:5000). Alexa Fluor™ 633-phalloidin (1:1000 for immunofluorescence staining, A22284) was also purchased from Invitrogen.

Chemicals were purchased from Sigma-Aldrich unless otherwise indicated. Jasplakinolide (2792) and 8-pCPT-2-O-Me-cAMP-AM (4853) were obtained from Tocris Bioscience (Minneapolis, MN) and dissolved in DMSO as per the manufacturer's instructions.

Mammalian expression plasmids pGFP and pGFP-cortactin were on the pGZ21dxZ vector backbone, pGFP plasmid was a kind gift from Dr Kenneth Yamada (NIDCR, NIH, Bethesda, MD), pGFP-cortactin was purchased from Addgene (plasmid #50728, deposited by Dr Kenneth Yamada). Plasmid DNA was purified using the QIAGEN Plasmid Midi Kit (12143) purchased from QIAGEN (Hilden, Germany) following the manufacturer's instructions.

### Animals

Female C57BL/6 mice at 6 to 8 weeks of age were purchased from Jackson Laboratories (Bar Harbor, ME) and were housed in a room with a 12-h light–12-h dark cycle with free access to water and rodent chow diet. The animals were inoculated intranasally with RSV A2 (3×10^5^ to 9×10^7^ pfu), UV-RSV, or an equal volume of supernatant from uninfected HEp-2 cells (control) on day 0 as described previously (Smallcombe et al., 2019). Mice were weighed daily and euthanized on day 4 post-inoculation, and lungs were harvested for immunohistochemistry staining.

All animal procedures used in this study adhered to the National Institutes of Health Guide for the Care and Use of Laboratory Animals and were reviewed and approved by the Institutional Animal Care and Use Committee (protocol 2018-2030) of the Lerner Research Institute at the Cleveland Clinic before the starting of experiments. This facility is accredited by the Association for the Assessment and Accreditation of Laboratory Animal Care (accreditation number 000383) and is following federal laws and NIH regulations.

### Cell culture

16HBE14o- (16HBE cells) were kindly provided by Dr Dieter Gruenert from the University of California, San Francisco, USA. This cell line was isolated from a 1-year-old male donor and immortalized with the origin-of-replication defective 158 SV40 plasmid. The cells were subjected to short tandem repeat (STR) analysis for cell line authentication and were tested negative for mycoplasma contamination (Callaghan et al., 2020) before usage. Cells were cultured under liquid–liquid conditions on tissue-treated T-75 flasks (CytoOne, Ocala, FL), multiple well plates (Corning, Tewksbury, MA), or collagen-coated Transwell-permeable inserts (Corning) until they were confluent (about 7 days after seeding). Type 1 rat tail collagen (354236, Corning) was used at 10 µl/ml. Cells were maintained in DMEM (11995, Gibco, Waltham, MA) supplemented with 0.5% antibiotic-antimycotic (15240-062, Gibco), 10% heat-inactivated fetal bovine serum (35-011-CV, Corning) and HEPES (15630, Gibco), and passed by trypsinization (25200056, Gibco). Experiments were performed using cell monolayers with a TEER>500 Ω cm^2^. Primary normal human bronchial epithelial (NHBE) cells (CC-2540, Lot 0000357048) were purchased from Lonza (Basal, Switzerland). NHBE cells were grown in defined medium and differentiated on Transwell inserts under air-liquid interface conditions as previously described (Rezaee et al., 2017a, 2011; Smallcombe et al., 2019). NHBE cells of passages 3–5 were used and were maintained for 4–5 weeks under air–liquid interface conditions before experiments with a TEER>1500 Ω cm^2^.

### RSV infection

Recombinant red fluorescent protein (RFP)-expressing recombinant RSV (rrRSV) derived from the RSV A2 strain was a kind gift from Dr Mark Peeples (Nationwide Children's Hospital Research Institute, Columbus, OH) and Dr. Peter Collins (National Institutes of Health, Bethesda, MD) (Smallcombe et al., 2020; Linfield et al., 2021a; Guerrero-Plata et al., 2006). RSV was grown and collected as previously described (Rezaee et al., 2013). Ultraviolet (UV)-inactivated RSV (UV-RSV) was obtained by exposing the virus to UVB radiation for 20 min. RSV infections of 16HBE cells were performed at MOI of 0.5 on monolayers 7 days after plating or otherwise described. RSV infections of NHBE cells were performed at MOI of 2 on differentiated monolayers.

### TEER

16HBE cells and NHBE cells were grown on Transwell-permeable inserts (3470, Corning) as described above, and then TEER was measured using the EVOMX volt-ohm meter (World Precision Instruments, Sarasota, FL) as previously described (Rezaee et al., 2011). Data were presented in percentage compared to indicated controls.

### Dextran permeability assay

Transmonolayer permeability was evaluated using fluorescein isothiocyanate (FITC)-conjugated dextran 4 kDa (46944, Sigma-Aldrich) as previously described (Smallcombe et al., 2020). Briefly, 16HBE cells were grown on Transwell-permeable inserts (3421, Corning) until they were confluent. The culture medium was removed from both the top and bottom chamber, then phosphate-buffered saline (PBS) was added to the bottom chamber, and FITC–dextran (540 µg/ml in PBS) was added to the top chamber. After incubation for 20 min at 37°C, samples were collected from the bottom chamber and FITC fluorescence was measured using FlexStation 3 (Molecular Devices, Sunnyvale, CA) at an excitation wavelength of 485 nm and an emission wavelength of 528 nm. Data were presented in fold compared to indicated controls.

### Immunofluorescent and immunohistochemistry staining

For immunofluorescent staining of F-actin and cortactin, cell monolayers on Transwell inserts were fixed in 4% paraformaldehyde (PFA) in PBS at room temperature for 5 min, rinsed three times with PBS, and incubated with 0.1% Triton X-100 in PBS at room temperature for 3 min. Cells were incubated with blocking buffer [5% bovine serum albumin (BSA) in PBS] for 1 h and then with primary antibodies in blocking buffer for 1 h at room temperature. After washing three times with PBS, cells were incubated with Alexa Fluor™-conjugated secondary antibody (1:500) for 1 h at room temperature and washed. Cells were stained with DAPI (1:1000; Sigma-Aldrich) for 5 min at room temperature, washed three times and mounted with VECTASHIELD mounting medium (H-1700, Vector Laboratories, Burlingame, CA). For immunofluorescent staining of TJ and AJ proteins, cell monolayers on Transwell inserts were fixed in cold methanol at −20°C for 20 min and washed with cold PBS three times. Cells were then incubated with blocking buffer and subjected to immunolabeling as described above. For immunohistochemistry staining, mouse lung tissues were stained as previously described (Smallcombe et al., 2019). Briefly, whole lungs were perfused, fixed in 4% PFA, and embedded in paraffin. Tissues were cut into 4-μm-thick sections, baked for 20 min at 65°C, deparaffinized and boiled in antigen retrieval buffer (10 mM trisodium citrate, 0.05% Tween-20, pH 6.0) for 30 min at 95°C. Tissue sections were blocked in 10% normal donkey serum with 0.1% Triton X-100 in PBS at room temperature for 1 h. Next, tissues were incubated overnight at 4°C with the primary antibodies, followed by incubation with Alexa Fluor™-conjugated secondary antibodies. Tissues were then stained with DAPI and mounted using Cytoseal XYL mounting medium (8312-4, Thermo Fisher Scientific, Waltham, MA). Immunolabeled samples were imaged with 40× or 63× objectives using a Leica TCS-SP8-AOBS inverted confocal microscope (Leica Microsystems, Wetzlar, Germany). Images of the apical surface were taken for cultured cells.

### Western blotting

Cells were washed twice with cold PBS and lysed on ice with RIPA buffer (89901, Thermo Fisher Scientific, Waltham, MA) supplemented with Halt™ protease and phosphatase inhibitor cocktail (1861281, Thermo Fisher Scientific). Samples were then centrifuged at 13,523 ***g*** at 4°C for 10 min to remove cell debris. Protein concentration was determined by the Pierce BCA Protein Assay kit (23225, Thermo Fisher Scientific). Proteins were denatured by adding 4× Laemmli sample buffer (161-0737, Bio-Rad Laboratories, Hercules, CA) and boiling for 5 min. Approximately 15 μg of total proteins were subjected to western blotting as previously described (Linfield et al., 2021a). Briefly, denatured samples were separated by SDS-PAGE and transferred to PVDF membranes (Bio-Rad Laboratories). Membranes were blocked in 5% BSA, probed with specific primary antibodies overnight at 4°C, and followed by HRP-conjugated secondary antibodies for 1 h at room temperature. Membranes were visualized with enhanced chemiluminescence (32106, Thermo Fisher Scientific) and imaged using the My ECL Imager imaging system (Thermo Fisher Scientific). Densitometry for each band was performed using LI-COR Image Studio™ Lite Software (Lincoln, NE) and values were normalized to controls as indicated. GAPDH was used as the loading control. Full-length blots showing the entire lanes with molecular mass markers were provided in Fig. S2.

### G-actin and F-actin fractionation assay

G-actin and F-actin fractions in cultured cells were separated using the G-Actin/F-Actin In Vivo Assay Kit according to the manufacturer's instructions (BK037, Cytoskeleton, Denver, CO). Briefly, cells were lysed using lysis and actin stabilization buffer supplemented with protease inhibitor cocktail and 1 mM ATP, scraped into Eppendorf tubes, and passed through a 25G syringe. Cell lysates were incubated at 37°C for 10 min and centrifuged at 350 ***g*** for 5 min to remove the debris. Supernatants were then transferred into ultracentrifuge tubes (349622, Beckman Coulter, Brea, CA) and centrifuged at 100,000 ***g*** at 37°C for 1 h. The resultant supernatants were designated as G-actin fractions. Then an equal volume of F-actin depolymerization buffer was added to the pellet and incubated on ice for 1 h with pipetting every 15 min. Resulting samples were designated as F-actin fractions. Each sample was then added 5× SDS sample buffer and boiled for 5 min to denature. An equal volume of samples from each fraction was loaded onto SDS-PAGE gels and subjected to western blotting. The amount of actin in G-actin or F-actin fraction was determined using a pan-actin antibody (A2103, Sigma-Aldrich) to detect all actin isoforms. Band intensity was quantified by densitometry and the G-actin:F-actin ratio was plotted.

### Transient transfection of 16HBE cells

16HBE cells were seeded onto Transwell-permeable inserts at a density of 5×10^4^ cells per well, 4 days (for cells grown on Transwell 3470, Corning) or 6 days (for cells grown on Transwell 3421, Corning) after passing, cells were transfected with Lipofectamine™ 3000 (L3000008, Invitrogen) according to manufacturer's instructions with modification. Briefly, 3 µg pGFP or pGFP-cortactin plasmid was mixed with 4.5 µl Lipofectamine™ 3000 and 4.5 µl P3000 in 40 µl Opti-MEM™ reduced-serum medium (31985062, Gibco). The resultant mixture was incubated for 15 min at room temperature before being added to the top of Transwell inserts. Medium from both the top and bottom of the Transwell was replaced with fresh medium 16 h after transfection. At 48 h after transfection, cells were subject to RSV infection at an MOI of 0.5. After 24 h of viral infection, cells were subjected to indicated experiments.

### Generation of cortactin KO 16HBE cells

The CRISPR/Cas9 system was used (sc-400761 and sc-400761-HDR, Santa Cruz Biotechnology, Dallas, TX) to knock out the human cortactin gene (*CTTN*) from 16HBE cells. 16HBE cells were seeded into six-well-plate at a density of 2×10^5^ cells per well, cells were transfected at approximately 50% confluency with Lipofectamine™ 3000 (L3000008, Invitrogen) according to the manufacturer's instructions. Briefly, 2 µg of cortactin CRISPR/Cas9 KO plasmid (sc-400761, Santa Cruz Biotechnology) and 2 µg of cortactin homology-directed DNA repair (HDR) plasmid (sc-400761-HDR, Santa Cruz Biotechnology) were mixed with 8 µl Lipofectamine™ 3000 and 8 µl P3000 in 250 µl Opti-MEM™ Reduced-Serum Medium (31985062, Gibco). The resultant mixture was added to the cells and the medium was changed 5 h after transfection. CRISPR/Cas9 KO Plasmid contains a pool of three guide RNAs plasmids targeting exons 3 and 12 of the human *CTTN* gene (Fig. S1A). The guide RNA sequences directed the Cas9 protein to induce a site-specific double-strand break in the genomic DNA, and the HDR template in the cortactin HDR plasmid directed the repair. When the *CTTN* gene was edited, an *RFP* gene and a puromycin resistance gene were inserted into the genome of the targeted cell. At 48 h after transfection, cells were selected with 1 µg/ml puromycin for 24 h to remove non-transfected cells. Then single cells were sorted into 96-well plates by fluorescence-activated cell sorting (FACS Melody™, BD Bioscience) using RFP as a selection marker. Single-cell colonies were grown for 1–2 weeks until they were confluent in the 96-well-plate, with the change of medium every other day. Next, the colonies were passed onto 24-well-plates and then 6-well-plates before validation. Single clones of cortactin KO cells were screened for deletion of cortactin by western blotting, and three clones were selected for further experiments.

To validate the occurrence of insertion-deletion mutations (indels) in single clones, genomic DNA was isolated using QuickExtract™ DNA extraction solution (QE0905T, Lucigen, Middleton, WI), and exon 3 or 12 of *CTTN* was amplified by PCR using the PrimeSTAR GXL DNA Polymerase (R050B, Takara Bio Inc., Kusatsu, Shiga, Japan) and primers listed in Table S2 (synthesized by Thermo Fisher Scientific). PCR amplicons were purified using the NucleoSpin Gel and PCR Clean-Up kit (740611.50, Takara Bio Inc.) and sequenced by Sanger sequencing. Results sequences were then analyzed by the Inference of CRISPR Edits analysis (Synthego Performance Analysis, ICE Analysis. 2019. v3.0., Synthego, Menlo Park, CA) (Hsiau et al., 2019 preprint). Mutations in both *CTTN* alleles were identified and frequencies of indel occurrence were evaluated.

### Rap1 activity assay

Rap1 activity was determined using the Rap1 activation kit (ab212011, Abcam) according to the manufacturer's instructions with modifications. Briefly, cells were washed twice with cold PBS and lysed on ice in the lysis buffer included in the kit. After lysis and centrifugation at 14,000 ***g*** for 10 min, supernatants were collected and whole-cell lysates were set aside. Rap1-GTP was pulled down by adding RalGDS-RBD-conjugated beads to the rest of the supernatant. After 2 h of incubation at 4°C, the beads were pelleted and washed six times with lysis buffer. The beads were finally resuspended in 40 μl of 2× SDS sample buffer and boiled for 5 min. Whole-cell lysates and pulldown samples were subjected to western blotting and immunoblotted with antibodies against Rap1.

### Data analysis and statistics

All data were generated from at least three biological replicates from independently prepared samples or experiments, the total number of biological replicates is provided in the figure legends. GraphPad Prism (version 8.1.1, GraphPad Software) was used for statistical analysis. Data are shown as mean±s.e.m. Data were analyzed by paired, two-tailed Student's *t*-test or one-way ANOVA (with Tukey's or Dunnett's post hoc test) as described. Statistical difference was considered when *P*<0.05. The *P* values are presented as *P<0.05, **P<0.01, ***P<0.001, with no significant difference indicated as ns.

## Supplementary Material

Supplementary information

Reviewer comments
