# Supplementary material for: Respiratory syncytial virus disrupts the airway epithelial barrier by decreasing cortactin and destabilizing F-actin
Source: J Cell Sci. 2022 Aug 16;135(16):jcs259871. doi: 10.1242/jcs.259871 (PMC9481929; doi:10.1242/jcs.259871)
Supplement: Supplementary information [file joces-135-259871-s1.pdf]

Fig. S1

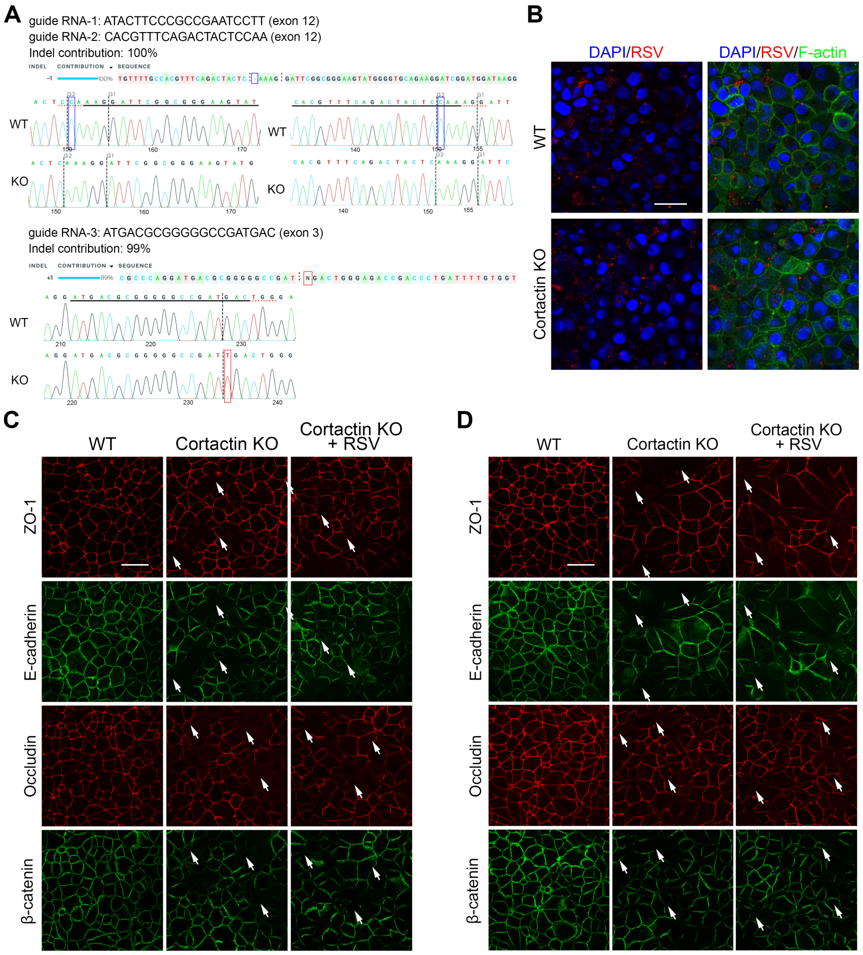
A
guide RNA-1: ATACTTCCCGCCGAATCCTT (exon 12)
guide RNA-2: CACGTTTCAGACTACTCCAA (exon 12)
Indel contribution: 100%
WT
KO
guide RNA-3: ATGACGCGGGGGCCGATGAC (exon 3)
Indel contribution: 99%
B
DAPI/RSV
DAPI/RSV/F-actin
WT
Cortactin KO
C
WT
Cortactin KO
Cortactin KO + RSV
ZO-1
E-cadherin
Occludin
β-catenin
D

Fig. S1. Related to Fig. 4. Disruptions in AJC staining are consistent across different clones of cortactin knockout 16HBE cells. (**A**) Sanger sequencing data and Inference of CRISPR Edits (ICE, Synthego) analysis results of *CTTN* exon 3 and exon 12 genomic region from a representative cortactin KO clone. The horizontal black underlined regions represent the guide RNA sequences. The horizontal red underlines indicate the PAM sequences. The vertical black dotted lines indicate the Cas9 cutting sites. The insertion-deletion (indel) types and percentages of detected sequence (contribution) are shown. Blue rectangles represent -1 indel mutations and red rectangles indicate +1 indel mutations. (**B**) Representative images of RSV virus 24h after inoculation indicated comparable RSV infection in WT and cortactin KO cells. Scale bar, 25 μm. (**C**) and (**D**) showed representative images from two independent clones that were different from the cells shown in Fig. 4. The structure of the apical junctional complex was determined by immunostaining with antibodies towards tight junction protein ZO-1 and occludin (red) as well as adherens junction protein E-cadherin and β-catenin (green). Arrows indicate disrupted AJCs in RSV-infected cells. Note that RSV infected-cortactin KO cells did not display further impairments in AJC structure compared with cortactin KO cells. Scale bar, 25 μm.

Journal of Cell Science • Supplementary information

Fig. S2

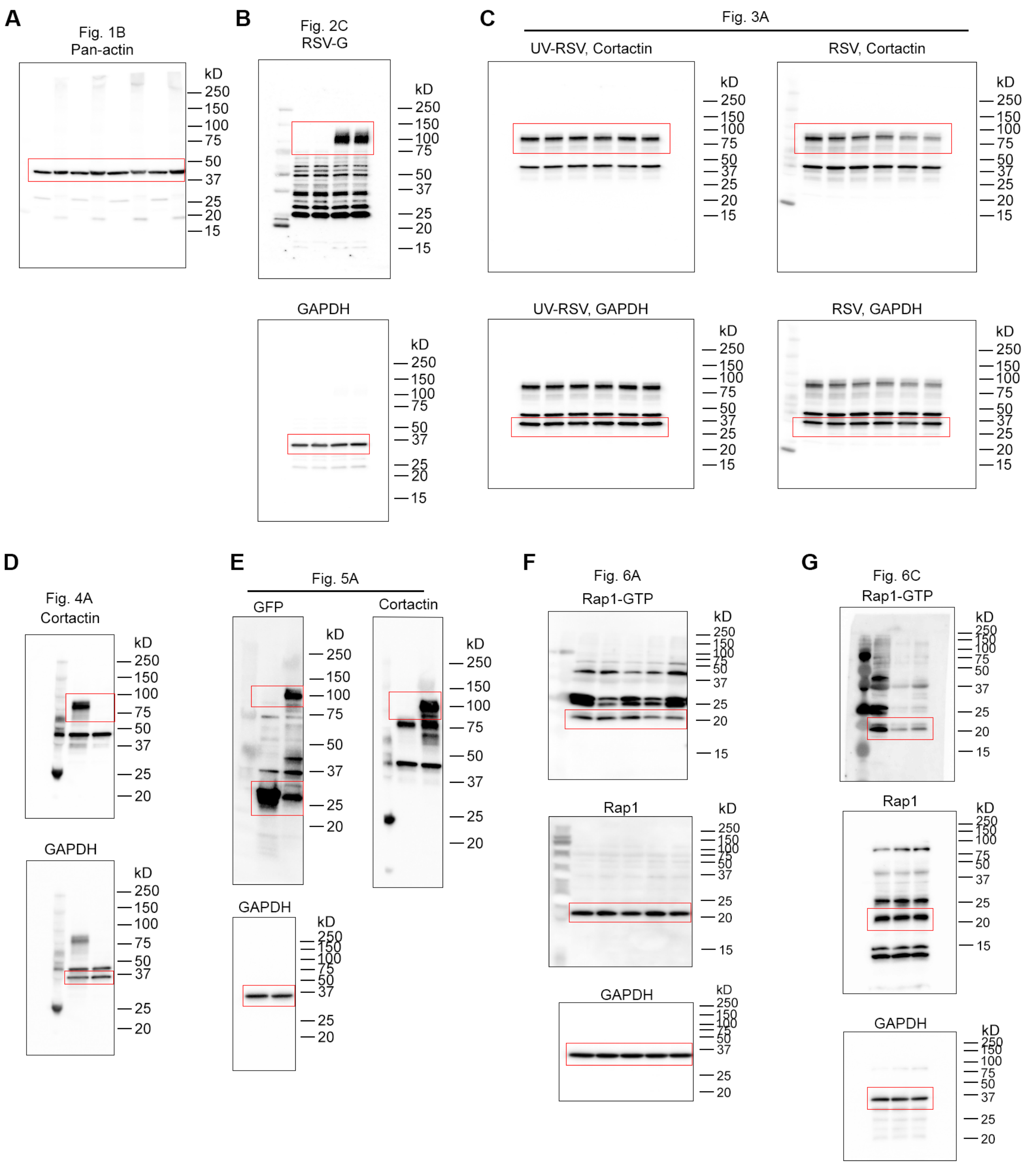

Fig. S2. Blot transparency. Full-length Western blots with indicated antibodies are shown for bands provided in Figures 1-6. Red rectangles are used to highlight where the bands were taken from.

Journal of Cell Science • Supplementary information

Table S1. Primary antibodies used for Western blot and immunostaining.

Antibodies	Source	Catalog number	Clone number	Validation	Working dilution
cortactin	Millipore Sigma	05-180	4F11	KO cell data included in this study (Fig. 4A)	1:1000 (WB)
cortactin	Abcam	ab33333	4F11	KO cell data included in this study (Fig. 4B)	1:300 (IF)
GAPDH	Abcam	ab8245	6C5	doi: 10.1038/s41467-018-08187-6	1:50,000 (WB)
pan-actin	Sigma-Aldrich	A2103	Polyclonal	doi: 10.3390/ijms21093152	1:1000 (WB)
ZO-1	Invitrogen	33-9100	1A12	doi: 10.1074/jbc.M114.556449	1:300 (IF)
ZO-1	Invitrogen	40-2200	polyclonal	doi: 10.3389/fncel.2012.00026; doi: 10.1074/jbc.M114.556449	1:300 (IF)
E-cadherin	Abcam	ab40772	EP700Y		1:300 (IF)
E-cadherin	BD Biosciences	610181	36	doi: 10.1371/journal.pgen.1008451	1:300 (IF)
E-cadherin	Cell Signaling Technology	3195S	24E10	doi: 10.1371/journal.pgen.1008451	1:100 (IHC)
occludin	Invitrogen	33-1500	OC-3F10	doi: 10.1248/bpb.b15-01023	1:300 (IF)
β-catenin	Abcam	ab32572	E247	doi: 10.1002/hep.30270	1:300 (IF)
RSV G protein (A2)	GeneTex	GTX70 381	polyclonal	doi: 10.1152/ajplung.00104.2020	1:1000 (WB)
GFP	Abcam	ab290	B-2	doi: 10.1016/j.celrep.2019.02.012	1:1000 (IF)
Rap1	Millipore Sigma	07-916	polyclonal	doi: 10.1016/j.celrep.2020.02.088	1:1000 (WB)

WB: Western blot
IF: immunofluorescence staining
IHC: immunohistochemistry staining

Table S2. Related to Fig. 4. PCR primers used to amplify targeted genomic DNA regions.

Human *CTTN* exon3:
Forward: 5’- GTGTTAGAACCCCGAGGTGA-3’
Reverse: 5’- ACCCCATCTTTGCTCCTTCT -3’

Human *CTTN* exon 12:
Forward: 5’- CATGGGTGGAAGCAAAACTT-3’
Reverse: 5’- GCACAAGACTGTCCGAGTCA-3’

Journal of Cell Science • Supplementary information
