# Supplementary material for: Respiratory syncytial virus disrupts the airway epithelial barrier by decreasing cortactin and destabilizing F-actin
Source: J Cell Sci. 2022 Aug 16;135(16):jcs259871. doi: 10.1242/jcs.259871 (PMC9481929; doi:10.1242/jcs.259871)
Supplement: Reviewer comments [file joces-135-259871_review_history.pdf]

Respiratory syncytial virus disrupts the airway epithelial barrier by decreasing cortactin and destabilizing F-actin

Nannan Gao, Andjela Raduka and Fariba Rezaee
DOI: 10.1242/jcs.259871

Editor: Michael Way

Review timeline

Original submission:	2 February 2022
Editorial decision:	15 March 2022
First revision received:	20 May 2022
Editorial decision:	24 June 2022
Second revision received:	8 July 2022
Accepted:	11 July 2022

Original submission

First decision letter

MS ID#: JOCES/2022/259871

MS TITLE: Respiratory syncytial virus disrupts airway epithelial barrier by decreasing cortactin expression and destabilizing F-actin

AUTHORS: Nannan Gao, Andjela Raduka, and Fariba Rezaee
ARTICLE TYPE: Research Article

We have now reached a decision on the above manuscript.

To see the reviewers' reports and a copy of this decision letter, please go to: https://submit-jcs.biologists.organd click on the 'Manuscripts with Decisions' queue in the Author Area. (Corresponding author only has access to reviews.)

As you will see, the reviewers raise a number of criticisms that prevent me from accepting the paper at this stage. They suggest, however, that a revised version might prove acceptable if you can address their concerns and comments. In particular, I think it is important that the level of RSV infection is determined in some of the key experiments as this point was raised by reviewer 2 and 3.If you think that you can deal satisfactorily with the criticisms on revision, I would be pleased to see a revised manuscript. We would then return it to the reviewers.

We are aware that you may be experiencing disruption to the normal running of your lab that makes experimental revisions challenging. If it would be helpful, we encourage you to contact us to discuss your revision in greater detail. Please send us a point-by-point response indicating where you are able to address concerns raised (either experimentally or by changes to the text) and where you will not be able to do so within the normal timeframe of a revision. We will then provide further guidance. Please also note that we are happy to extend revision timeframes as necessary.

Please ensure that you clearly highlight all changes made in the revised manuscript. Please avoid using 'Tracked changes' in Word files as these are lost in PDF conversion.

© 2022. Published by The Company of Biologists under the terms of the Creative Commons Attribution License (https://creativecommons.org/licenses/by/4.0/).

I should be grateful if you would also provide a point-by-point response detailing how you have dealt with the points raised by the reviewers in the 'Response to Reviewers' box. Please attend to all of the reviewers' comments. If you do not agree with any of their criticisms or suggestions please explain clearly why this is so.

Reviewer 1

Advance summary and potential significance to field

In this manuscript, Gao and colleagues show that RSV induces epithelial barrier breakdown via cortactin downregulation and actin cytoskeleton depolymerization. These responses are related to reduced Rap1 activation as a consequence of reduced cortactin expression. The mechanism per se is not novel as it has been shown previously in other epithelial and endothelial cells that the absence of cortactin reduces Rap1 activation and alters actin dynamics. However, in the context of RSV infection this has not been shown before; and it is important to see that this mechanism occurs in different settings and is thus of general cell biological importance. Thus, I consider the data interesting and of relevance for the broad readership of JCS. However, it will be important to better relate these new data to the existing literature.

Comments for the author

In endothelial and intestinal epithelial cells cortactin depletion was accompanied by increased stress fiber formation. Why is that not seen in when cortactin is reduced in response to RSV infection. Cortactin depletion has been shown to not only cause reduced Rap1 activity, but also increased RhoA activity that in turn caused increased ROCK1-mediated MLC phosphorylation and actomyosin contractility. The infected cells here do not show more fibers. Thus, what additional mechanisms downstream of the virus could be at play that induces only actin depolymerization (increased G-actin) and prevents stress fiber formation? What happens to Rac1, cdc42 and RhoA after infection that causes cortactin depletion? Most small GTPases are actually affected somehow by the absence of cortactin (see also Lai et al 2009 Mol Biol Cell).
Also, the very recent article by Hunziker et al. Cell Reports, 2022 needs to be included and discussed properly as it is directly related to this study, although they used a different virus. In this study, they found different cortactin phosphorylation patterns in response to IAV that led to cortactin recruitment to sites of infection and altered cdc42-dependent filopodia dynamics. Cortactin expression restricted virus entry. In this sense, the observed downregulation here makes sense to also facilitate RSV entry. But it would be nice, if the authors could test if the remaining cortactin also gets differently phosphorylated in response to RSV infection. Also, why is no relocalization observed after RSV infection in contrast to IAV infection. These are important points that need to be at least discussed very critically.
In Figure 5 it will be important to include permeability measurements in cortactin KO cells. If the effects are really related, as suggested, no additive effect should be seen. This also applies to figure 7, is there no additive effect on Rap1 activity reduction in RSV-infected KO cells?
In figure 6, the rescue effect on TEER by cortactin-GFP is rather minor while the rescue effect on flux is almost complete. Why is that? The statement in lines 215-217 is not really supported by the figure; intact ZO-1 and E-cadherin staining are also seen around GFP-negative cells. More convincing images are required to maintain this conclusion.
It does not seem that cortactin-GFP gets degraded after infection (6E). Does the GFP mask the site required for virus-induced cortactin degradation? Any ideas what the degradation mechanism is? Or is it rather reduced de novo production? While it is certainly beyond the scope of this manuscript to identify a degradation mechanism, a simple PCR could reveal whether cortactin mRNA production is reduced after infection that would consequently cause reduced protein levels.
Minor points Figure 3 could be merged with Figure 2 as the findings are related.
Although in general well-written, proof-reading is recommended to eliminate some errors throughout the text.

© 2022. Published by The Company of Biologists under the terms of the Creative Commons Attribution License (https://creativecommons.org/licenses/by/4.0/).

<u>Reviewer 2</u>

Advance summary and potential significance to field

Gao et al. described respiratory syncytial virus (RSV) induced epithelial barrier disruption by decreasing cortactin expression and destabilizing F-actin. However, their claim about RSV-induced F-actin destabilization in airway epithelial cells is not convincing. It is important to note that RSV detection was done in experiments neither in vitro nor in vivo. Therefore, the detection of RSV-infected cells and determination of the infection rate are important before concluding.

Overall, the authors suggested RSV infection causes barrier disruption via actin depolymerization. Cortactin has N-terminal acidic domain which interacts with ARP2/3 complex leading to actin filamentous branching and network formation (Schnoor et al., 2018). However, they did not compare or describe whether RSV induces syncytium or modulates ARP2/3 complex-driven actin polymerization (F-actin) in 16HBE cells, as RSV-induced F-actin depolymerization wasn't clear in the results (see comments). Mehedi et al., 2016 showed ARP2 and virus-induced filopodia facilitate RSV spread in A549 cells.

Notably, the authors did not use a correct F-actin depolymerization inhibitor (see manjor concerns). Thus RSV-induced F-actin depolymerization and cortactin's contribution (detection of RSV infected cells) to the epithelial barrier destabilization must be established in an in vitro model, preferably ALI culture (at least 21 days differentiation).

Based on the above description, this reviewer thinks the maunscript does not advanceor conteribute to the relavent field or research.

Comments for the author

This reviwer think this manuscript is not suitbale for publication. The reasonse are descrbed below. Gao et al. described respiratory syncytial virus (RSV) induced epithelial barrier disruption by decreasing cortactin expression and destabilizing F-actin. However, their claim about RSV-induced F-actin destabilization in airway epithelial cells is not convincing. It is important to note that RSV detection was done in experiments neither in vitro nor in vivo. Therefore, the detection of RSV-infected cells and determination of the infection rate are important before concluding.
Overall, the authors suggested RSV infection causes barrier disruption via actin depolymerization. Cortactin has N-terminal acidic domain which interacts with ARP2/3 complex leading to actin filamentous branching and network formation (Schnoor et al., 2018). However, they did not compare or describe whether RSV induces syncytium or modulates ARP2/3 complex-driven actin polymerization (F-actin) in 16HBE cells, as RSV-induced F-actin depolymerization wasn't clear in the results (see comments). Mehedi et al., 2016 showed ARP2 and virus-induced filopodia facilitate RSV spread in A549 cells.
Notably, the authors did not use a correct F-actin depolymerization inhibitor (see manjor concerns). Thus RSV-induced F-actin depolymerization and cortactin's contribution (detection of RSV infected cells) to the epithelial barrier destabilization must be established in an in vitro model, preferably ALI culture (at least 21 days differentiation).

Major comments:
1.The authors suggested that RSV A2 strain infection at a MOI=0.5 reduced F-actin staining at 24-hour post-infection (Fig 1A). Without detecting virus-infected cells and determining the percentage of viral infection, it is impossible to understand the results of Figure 1A and subsequent figures. Does RSV infection reduce F-actin only in infected cells or all cells? Figure 1A does not clarify whether all cells were infected by RSV and reduced F-actin. The author can utilize high-resolution imaging (confocal or FRET) to determine RSV-induced F-actin depolymerization at a single-cell level.
2.For Figure 1B, cells were treated with actin depolymerization inhibitor for 2 hours after 22-hour RSV-infection to restore F-actin in the infected cells. Here, detection of virus-infected cells and determination of the percentage of viral infection are required to determine F-actin restoration in all cells or RSV-infected cells by the jasplakinolide.
3.Jasplakinolide is an inducer of actin polymerization but not an inhibitor of actin depolymerization. Indeed reference 51 (Bobb M.R. et al, 1994, JBC 269) suggests "Actin filament

© 2022. Published by The Company of Biologists under the terms of the Creative Commons Attribution License (https://creativecommons.org/licenses/by/4.0/).

made by the addition of jasplakinolide were resistant to depolymerization". Therefore, experimental results with jasplakinolide (Figures 1-3) need to revisit with a correct F-actin depolymerization inhibitor.
4.Figure 2, Without viral infection rate (and detection of infected cells) it is difficult to understand the results.
For example, RSV is known to form syncytia in vitro, no syncytia formation in vivo or more appropriate in vitro model, e. g., ALI culture. It needs to be determined whether syncytia formation disrupted cell-cell junction.
RSV may cause syncytia formation in the 16HBE cell line. Then the syncytia formation is more likely an irreversible event. It must be determined whether the F-actin depolymerization inhibitor can restore RSV-induced CPE, increasing barrier impermeability.
5.RSV infection causes cytopathic effect (CPE) on infected cell monolayer. In Fig 4A&B, the observed less cortactin could be the result of the RSV-induced cell death. Besides, UV-RSV is not an appropriate control.
Therefore, RSV-induced cortactin reduction should be established at a single-cell level.
6.Figure 4E shows that the RSV-infection rate or RSV infection needs to be established in the ALI. Additionally, RSV-induced F-actin depolymerization hasn't been established in the ALI.
7.Figure 4 F-H, the results are difficult to interpret without RSV detection in the infected epithelium.
8.Figure 5-6, Cortactin involvement needs to be established in the RSV infection before further characterization of cortactin in cells

Minor comments:
1.Fig 4E, Orthogonal (XZ or YZ) view will help to observe the respiratory epithelium (Here, only XY plane was presented). Moreover, it is not specified which Z-plane was taken for this image preparation. The result is not convincing without displaying RSV infection in the apical surface. Again, RSV-infected cells are not detected here.
2.According to reference 11, the authors measured TEER just 7-day post-seeding in ALI culture but did not show what the epithelium looked like during that time. Based on the TEER measurements of previous reports it can assume that the epithelial cell barrier will not be functional enough in ALI culture during 7-day post-seeding.
4.According to reference 11 (also used in Reference 10 and 30), air-liquid interface (ALI) culture was maintained for only 14 days. Multiple groups (Michi et al., 2021, Broadbent et al., 2020, and Osan et al., 2021) suggested ALI culture needs differentiation at least 3 weeks; however, 4 weeks is preferable. The higher differentiation tends to mimic the biophysical properties of in vivo airways.

Reviewer 3

Advance summary and potential significance to field

The authors of this study demonstrate convincingly that infections of (airway) epithelial cells with Respiratory syncytial virus(RSV) reduces the expression of cortactin followed by accompanied downstream effects previsouly established by others in different systems, such as compromized Rap/Rac signaling and epithelial junction destabilization. The authors also show that their airway epithelial cell model displays phenotypes comparable to RSV infections if cortactin expression is eliminated by CRISPR/Cas9. The reduction of cortactin expression effected by RSV infection requires replicative activity of the virus but the mechanistic connection between viral replication and downregulation of cortactin expression remains unknown.
This is a quite complete study that complements previous knowledge both on RSV biology and cellular as well as organismic cortactin functions. The only major experiment missing in my view was to perform RSV infections on the cortactin-depleted 16HBE cells shown and characterized in Fig. 5. Given that RSV can still enter the cells (which I assume but would have to be clearly demonstrated), this experiment would allow the authors to judge or estimate at least the relative contribution that the downregulation of cortactin is playing alongside with potential additional changes caused by RSV infection. If the observed downregulation of cortactin expression by RSV has a significant contribution to the phenotypes developed upon RSV infections (which is assumed at present and the main conclusion of the study), the extent of additional effects caused by RSV in the absence of cortactin should be eliminated or at least reduced. This applies in particular to the reduction of TEER and increase of dextran flux already observed in the cortactin null cell line (Figure 5), but also, of course, to the partial disintegration of epithelial junctions, as judged by

© 2022. Published by The Company of Biologists under the terms of the Creative Commons Attribution License (https://creativecommons.org/licenses/by/4.0/).

ZO-1, E-cadherin, occluding or beta-catenin staining. In conclusion, in case this experiment is provided and displays a clear outcome (whatever this may be), I would strongly vote for publication of the study in due time.
Concerning the Figures, in particular in the context of potential addition of data. The figures are nice and well understandable, even without much reading of the legends (I much like the model in Figure 9 – this is beautiful), but if additional space is required, some of them could certainly be combined or moved to the Supplement (with a specific suggestion given below).

Comments for the author

There are only a few important points from my side at this stage that read as follows:
1) I am not sure how relevant Figure 3 is as stand alone really. This could be combined with the previous Figure or moved to the supplement.
2) The headline of Fig. 5 reads…"Epithelial barrier integrity is disrupted in cortactin knockout…". Looking at the data, "disrupted" seems a bit strong, as I feel that the effect at best constitutes a partial weakening and "thickening"
of junctional linings between cells (this is at least how it appears in most cells at the currently shown magnification). Given previous reports in the literature, I assume the apparent thickening constitutes a more disorganized zig-zag arrangement of the junctional connections between cells, but no complete disruption. This could be a bit confusing to readers, so in general, the images shown concerning junctional integrity in different treat6ments in the paper (RSV infections and cortactin-KO) could be complemented by higher mag images clearly defining what's going on. This would probably also work if selecting just one condition, like the cortactin KO, which will likely give the cleanest and clearest phenotype in this respect, as representative example – but this will of course be up to the authors.
3) Concerning the characterization of CRISPR-Cas9- mediated cortactin gene disruption. This is nice, and I understand the authors to have generated individual KO clones. However, it remained unclear to me when briefly glancing through the Methods section whether the authors are showing data for just one clone in Figure 5 or a mixture of clones perhaps, and whether they can recapitulate phenotypes in several, optimally up to 3-5 clones, which would really exclude non-specific, off-target effects in my view. Another possibility of excluding off-target effects (which I do not find likely here personally though) could be to rescue the phenotypes with GFP-cortactin used in the experiment in Figure 6, but this might be asking for a bit much because possibly technically challenging. So instead, I would recommend providing a Supplementary Figure with results from a few independently generated cortactin null clones which could very well complement or replace the need for a rescue. However, I have also just noticed that the authors have refrained from providing any information on how the cortactin null clones were characterized at the genomic level. I would strongly recommend performing TIDE sequencing (or alike, see e.g. PMID: 25300484) of a few CRISPR-clones selected based on Western data, allowing to examine the precise mutations introduced and their frequency of occurrence as well as potentially excluding the presence of any wildtype alleles in individual clones.
4) I did not understand why Figure 6A shows an admittedly less abundant but still clearly detectable band at the molecular size of GFP in the GFP-cortactin sample on the right, although solely transfected with GFP-cortactin I guess.
This is probably not uncommon though, but likely suggests degradation of ectopic cortactin down to the size GFP. This should not be left uncommented I think, and I would also encourage showing entire blots (at least in the Supplement), so that the reader can appreciate the extent of GFP-cortactin degradation in this experiment. Indeed, the extent of improvement of TEER in GFP-cortactin-transfected, RSV-infected cells was statistically significant, yet quite limited
(as compared to the extent of decrease of FITC-dextran flux, for instance), so this fact might be explained, at least in part, by quite strong GFP-cortactin degradation. Whatever the case, additional information in this context might give the reader the chance at least to judge themselves how all these data fit together.
5) As already elaborated on in the general comments above, the most significant experiment in my view concerning this story would be to take a well-
characterized, cortactin-deficient clone and explore the extent of changes still induced by RSV infections!

© 2022. Published by The Company of Biologists under the terms of the Creative Commons Attribution License (https://creativecommons.org/licenses/by/4.0/).

First revision

Author response to reviewers' comments

Reviewer 1 Advance Summary and Potential Significance to Field...

In this manuscript, Gao and colleagues show that RSV induces epithelial barrier breakdown via cortactin downregulation and actin cytoskeleton depolymerization. These responses are related to reduced Rap1 activation as a consequence of reduced cortactin expression. The mechanism per se is not novel as it has been shown previously in other epithelial and endothelial cells that the absence of cortactin reduces Rap1 activation and alters actin dynamics. However, in the context of RSV infection this has not been shown before; and it is important to see that this mechanism occurs in different settings and is thus of general cell biological importance. Thus, I consider the data interesting and of relevance for the broad readership of JCS. However, it will be important to better relate these new data to the existing literature.

Reviewer 1 Comments for the Author...

In endothelial and intestinal epithelial cells cortactin depletion was accompanied by increased stress fiber formation. Why is that not seen in when cortactin is reduced in response to RSV infection. Cortactin depletion has been shown to not only cause reduced Rap1 activity, but also increased RhoA activity that in turn caused increased ROCK1-mediated MLC phosphorylation and actomyosin contractility. The infected cells here do not show more fibers. Thus, what additional mechanisms downstream of the virus could be at play that induces only actin depolymerization (increased G-actin) and prevents stress fiber formation? What happens to Rac1, cdc42 and RhoA after infection that causes cortactin depletion? Most small GTPases are actually affected somehow by the absence of cortactin (see also Lai et al 2009 Mol Biol Cell).

Response:

1. Our explanation regarding the observations that RSV infection induced actin depolymerization but did not cause stress fiber formation in our manuscript is as follows.

   While cells grow and become confluent, cell-cell junction formation is accompanied by rearrangements in the actin cytoskeleton. When cells are solitary and not organized into tightly packed sheets, they are flat and form focal contacts with the extracellular matrix where the actin stress fibers attach. In contrast, cells take a columnar shape in a fully confluent epithelial sheet, and a cortical belt of F-actin cables underlies the intercellular junctions at the apical side. This integrated actin network span throughout the epithelial sheet and exhibits a honeycomb pattern in an apical view, as we showed in Figure 1, while the classic focal adhesions and actin stress fibers are localized at the basal plane of the monolayer. The changes in actin dynamics and cytoskeletal polarization during the epithelial sheet formation have been beautifully revealed utilizing GFP-Actin transgenic mice (Vaezi A et al., Dev Cell, 2002). The immunostaining of F- actin in this study was imaged at the apical plane of the monolayer, while the G-actin/F-actin ratio provided an estimation of the cellular F-actin network as a whole. We did not directly examine the stress fiber at the basal side in RSV-infected 16HBE monolayers. However, our group previously found that RSV did induce stress fiber formation in detached and reseeded 16HBE cells and attenuate cell migration after wounding by increasing RhoA and phospho-myosin light chain 2 (MLC2) (Linfield DT et al., Am J Physiol Lung Cell Mol Physiol, 2021), so it’s likely that the decrease of cortactin contributes to stress fiber formation in cells at this non-confluent state.
   Collective, these data imply that the effect of RSV infection-induced cortactin reduction on actin cytoskeleton depends on the states of cells (confluent or solitary) and needs to be interpreted in a context-specific manner.

2. As for changes in other small GTPases after RSV infection, our preliminary data on the activity of Rac1 in RSV-infected cells did not reveal a significant change.

© 2022. Published by The Company of Biologists under the terms of the Creative Commons Attribution License (https://creativecommons.org/licenses/by/4.0/).

[NOTE: We have removed a figure which was provided for the referees in confidence.]

> As we pointed out above, we observed increased RhoA and MLC2 phosphorylation following RSV infection in a wound model using16HBE cells (Linfield DT et al., Am J Physiol Lung Cell Mol Physiol, 2021), however, we didn't detect a significant change in pMLC2/MLC2 level using western blot in RSV-infected non-wounded cells, suggesting that RhoA/pMLC2 pathway may not change significantly after RSV infection in naïve 16HBE monolayers. The underlying mechanisms leading to such discrepancies warrant further investigation.

Also, the very recent article by Hunziker et al. Cell Reports, 2022 needs to be included and discussed properly as it is directly related to this study, although they used a different virus. In this study, they found different cortactin phosphorylation patterns in response to IAV that led to cortactin recruitment to sites of infection and altered cdc42-dependent filopodia dynamics. Cortactin expression restricted virus entry. In this sense, the observed downregulation here makes sense to also facilitate RSV entry. But it would be nice, if the authors could test if the remaining cortactin also gets differently phosphorylated in response to RSV infection. Also, why is no relocalization observed after RSV infection in contrast to IAV infection.
These are important points that need to be at least discussed very critically.

Response:
Thank you for these insightful comments. We added a discussion of *Hunziker et al. Cell Reports, 2022* on page 15, lines 367-379 in our updated manuscript.

As for the change in phosphorylation of the remaining cortactin after RSV infection, we found a robust decrease at the whole-cell level, which in our opinion might cause impacts as profound as an altered post-translational modification if not greater. On the other hand, cortactin contains multiple phosphorylation sites, which complicates investigation using site-specific antibodies, so analyzing the phosphorylation pattern like Hunziker et al can be expensive and time-consuming for us. Although we recognize that this question is interesting and could be an important general mechanism among different viral infections, it is unfortunately out of the scope of our current study but provides a direction for future research.

About the absence of cortactin relocalization after viral infection reported in *Hunziker et al. Cell Reports, 2022*, we did not focus on the first 10-20min within RSV infection because our interest is the disruption of the barrier rather than virus entry, the change in cortactin relocation might be a time frame-sensitive phenomenon and therefore was not captured by our experiments.

In Figure 5 it will be important to include permeability measurements in cortactin KO cells. If the effects are really related, as suggested, no additive effect should be seen. This also applies to figure 7, is there no additive effect on Rap1 activity reduction in RSV-infected KO cells?

Response: Thank you for this great advice. As suggested, we examined the barrier permeability in RSV- infected cortactin KO cells. Indeed, through measurements of TEER, dextran efflux assay, and immunostaining towards the apical junctional complex, we did not observe significant additional impacts on the barrier function from RSV in KO cells (Figure 4C-F). These data suggest that cortactin is a major downstream factor mediating RSV-induced barrier disruption. We have included the description of these results on page 10 lines 232-235. In addition, we assessed the activity of Rap1 in RSV-infected KO cells, and no significant additive decrease in Rap1 activity was observed compared to KO cells (Figure 6C, 6D). These data have now been included in our updated manuscript and explained on pages 11-12 lines 275- 280.

In figure 6, the rescue effect on TEER by cortactin-GFP is rather minor while the rescue effect on flux is almost complete. Why is that? The statement in lines 215-217 is not really supported by the figure; intact ZO-1 and E-cadherin staining are also seen around GFP-negative cells. More convincing images are required to maintain this conclusion.
It does not seem that cortactin-GFP gets degraded after infection (6E). Does the GFP mask the site required for virus-induced cortactin degradation? Any ideas what the degradation mechanism is? Or is it rather reduced de novo production? While it is certainly beyond the scope of this manuscript to

© 2022. Published by The Company of Biologists under the terms of the Creative Commons Attribution License (https://creativecommons.org/licenses/by/4.0/).

identify a degradation mechanism, a simple PCR could reveal whether cortactin mRNA production is reduced after infection that would consequently cause reduced protein levels.

Response: Thank you for bringing these questions to our attention.

1. Regarding the limited improvement of TEER by overexpressing GFP-cortactin and the discrepancy between the rescues effect of GFP-cortactin on TEER and dextran flux, we'd like to provide more information on the differences between these two parameters. The electrical resistance and the permeation of macromolecule tracers such as dextran (we used 4 kD dextran in this study) are both indicators of paracellular permeability, electrical resistance is a measurement of the transjunctional flow of small inorganic molecules (mostly ions), while the dextran permeability characterizes the flux of high molecular weight biomolecules across the cellular barrier (reviewed in detail by Radoslaw Bednarek, Methods Protoc., 2022). Therefore, results from these assays are not linearly correlated and their degree of correlation worsens as the molecular size of the solute increases (Madara, J.L. & Dharmsathaphorn, K. J. Cell Biol. 1985). In our study, the reflection of restored barrier integrity by the decrease in FITC- dextran flux was more noticeable than the improvement in TEER, probably because of the size differences between the molecules they measured. We have updated our manuscript to include this additional information in the Discussion (page 16, lines 395-402).

2. About the statement in lines 215-217 in the previous version of the manuscript, based on our observation and images provided in this manuscript, immunostaining images of the barrier disruption caused by RSV are not homogeneous and exhibit a 'patchy' pattern, this makes it difficult to precisely sort out the improvement effect of GFP-cortactin on a single-cell level. Therefore we rephrased the description in the updated manuscript (page 11, lines 252-254)

3. For Figure 6E, the GFP-cortactin staining data we presented was not direct imaging of the GFP: for best results of the immunostainings against TJ/AJ proteins, we fixed cells with cold methanol at -20°C, which would denature or "quench" the GFP and thus made the direct imaging of GFP unreliable, or even infeasible. Therefore, to visualize GFP-cortactin as an indication for transfection, we stained our samples with anti-GFP primary antibody and Alexa Fluor 488 secondary antibody. Doing so might have complicated one's observation regarding the change in overexpressed cortactin because the GFP signals could be a mixture of both direct fluorescence and antibody staining. However, we included a full-length immunoblot of cell lysates using GFP antibodies, in which the lower molecular weight displayed degradation of cortactin (Supplementary Figure 2E).

   As for the possible degradation mechanism, we performed a real-time PCR experiment as the reviewer suggested. Results from two different pairs of primers revealed that the mRNA level of cortactin did not alter significantly after RSV infection, indicating that the observed decrease in the protein level of cortactin could be the consequence(s) of post-transcriptional and/or post- translational regulations. This would be an interesting question to pursue in the future.

[NOTE: We have removed a figure which was provided for the referees in confidence.]

Minor points
Figure 3 could be merged with Figure 2 as the findings are related.

Response: Thank you for this helpful feedback, we merged Figure 3 with Figure 2 in the updated manuscript (now Figure 2 in the updated manuscript).

Although in general well-written, proof-reading is recommended to eliminate some errors throughout the text.
Response: Thank you for this suggestion. We conducted proofreading of this manuscript, and errors have been corrected.

© 2022. Published by The Company of Biologists under the terms of the Creative Commons Attribution License (https://creativecommons.org/licenses/by/4.0/).

Reviewer 2 Advance Summary and Potential Significance to Field...

Gao et al. described respiratory syncytial virus (RSV) induced epithelial barrier disruption by decreasing cortactin expression and destabilizing F-actin. However, their claim about RSV-induced F-actin destabilization in airway epithelial cells is not convincing. It is important to note that RSV detection was done in experiments neither in vitro nor in vivo. Therefore, the detection of RSV-infected cells and determination of the infection rate are important before concluding.

Overall, the authors suggested RSV infection causes barrier disruption via actin depolymerization. Cortactin has N-terminal acidic domain which interacts with ARP2/3 complex leading to actin filamentous branching and network formation (Schnoor et al., 2018). However, they did not compare or describe whether RSV induces syncytium or modulates ARP2/3 complex-driven actin polymerization (F-actin) in 16HBE cells, as RSV-induced F-actin depolymerization wasn't clear in the results (see comments).
Mehedi et al., 2016 showed ARP2 and virus-induced filopodia facilitate RSV spread in A549 cells.

Notably, the authors did not use a correct F-actin depolymerization inhibitor (see manjor concerns). Thus RSV-induced F-actin depolymerization and cortactin's contribution (detection of RSV infected cells) to the epithelial barrier destabilization must be established in an in vitro model, preferably ALI culture (at least 21 days differentiation).

Based on the above description, this reviwer thinks the maunscript does not advanceor conteribute to the relavent field or research.

Reviewer 2 Comments for the Author...

This reviwer think this manuscript is not suitbale for publication. The reasonse are descrbed below.

Gao et al. described respiratory syncytial virus (RSV) induced epithelial barrier disruption by decreasing cortactin expression and destabilizing F-actin. However, their claim about RSV-induced F-actin destabilization in airway epithelial cells is not convincing. It is important to note that RSV detection was done in experiments neither in vitro nor in vivo. Therefore, the detection of RSV-infected cells and determination of the infection rate are important before concluding.

Overall, the authors suggested RSV infection causes barrier disruption via actin depolymerization. Cortactin has N-terminal acidic domain which interacts with ARP2/3 complex leading to actin filamentous branching and network formation (Schnoor et al., 2018). However, they did not compare or describe whether RSV induces syncytium or modulates ARP2/3 complex-driven actin polymerization (F-actin) in 16HBE cells, as RSV-induced F-actin depolymerization wasn't clear in the results (see comments).
Mehedi et al., 2016 showed ARP2 and virus-induced filopodia facilitate RSV spread in A549 cells.

Notably, the authors did not use a correct F-actin depolymerization inhibitor (see manjor concerns). Thus RSV-induced F-actin depolymerization and cortactin's contribution (detection of RSV infected cells) to the epithelial barrier destabilization must be established in an in vitro model, preferably ALI culture (at least 21 days differentiation).

Major comments:

1. The authors suggested that RSV A2 strain infection at a MOI=0.5 reduced F-actin staining at 24- hour post-infection (Fig 1A). Without detecting virus-infected cells and determining the percentage of viral infection, it is impossible to understand the results of Figure 1A and subsequent figures. Does RSV infection reduce F-actin only in infected cells or all cells? Figure 1A does not clarify whether all cells were infected by RSV and reduced F-actin. The author can utilize high-resolution imaging (confocal or FRET) to determine RSV-induced F-actin depolymerization at a single-cell level.

Response: Thank you for the great advice.

© 2022. Published by The Company of Biologists under the terms of the Creative Commons Attribution License (https://creativecommons.org/licenses/by/4.0/).

To address the reviewer's question about the percentage of viral infection, we performed confocal imaging and analysis. The recombinant RSV A2 strain used in this study was modified to express the red fluorescent protein (RFP) upon replication (please see Materials and Methods session, pages 18-19, line 466-475), therefore we imaged the RFP signals as an indication of RSV infection and analyzed the percentage of RFP-positive 16HBE cells. Here, panel A shows a representative image for cells 24 hours after infection of RSV at an MOI of 0.5 (Scale bar, 30 μm). Statistical results showed that RSV caused infections in about 63% of 16HBE cells under the aforementioned condition (B, n = 10 images, each image contains 70-120 cells).

[NOTE: We have removed a figure which was provided for the referees in confidence.]

Regarding the second point, to characterize RSV-induced F-actin depolymerization at a single-cell level, we determined the mean fluorescence intensity of F-actin in RSV-infected cells and RSV-uninfected cells. Compared with control cells, the average intensity of F-actin declined in RSV-infected cells (to 70.2% of control cells); moreover, uninfected cells also displayed a reduction in F-actin intensity (to 76.8% of control cells), which was comparable to that observed in infected cells (n = 50 cells from 10 images). Our data suggest that RSV infection reduces F-actin in both infected cells and uninfected cells on the same transwell insert. One possible explanation for this phenomenon is the inflammatory response caused by RSV infection. It is well documented that RSV triggers the expression and secretion of cytokines and chemokines from airway epithelial cells (Noah T. L. and Becker S., Am J Physiol Lung Cell Mol Physiol, 1993; Zhang Y., J Virol, 2001; Smallcombe CC et al., Am J Physiol Lung Cell Mol Physiol, 2020), and the resulted release of cytokines such as IL-4, IL-13, and IFNγ has been shown to disrupt epithelial barrier integrity (Bruewer M et al., FASEB J, 2005; Utech M et al., Mol Biol Cell, 2005; Saatian B et al., Tissue Barriers, 2013). Therefore, the observed effects of RSV infection in our model could reflect both a direct effect of viral infection and a consequence of released inflammatory cytokines and chemokines in response to viral infection.

[NOTE: We have removed a figure which was provided for the referees in confidence.]

2. For Figure 1B, cells were treated with actin depolymerization inhibitor for 2 hours after 22-hour RSV- infection to restore F-actin in the infected cells. Here, detection of virus-infected cells and determination of the percentage of viral infection are required to determine F-actin restoration in all cells or RSV-infected cells by the jasplakinolide.

Response: Thank you for your suggestion and we recognize that it is essential to establish the percentage of viral infection in our model. Similar to what we did in response to comment 1, we imaged the RFP signals as an indicator of RSV infection and analyzed the percentage of RFP-positive 16HBE cells described in Figure 1B. Statistical results showed that the RSV infection ratio in 16HBE cells was comparable in RSV infected cells treated with vehicle versus cells treated with jasplakinolide (55.3% in RSV + vehicle group and 57.8% in RSV + jasp. group, n = 5 images, each image contains 50-80 cells). Additionally, western blot against RSV-G protein also indicated that RSV infection did not change significantly between these two groups (Figure 2C-D). In our response to comment 1, we showed that RSV infection led to F-actin depolymerization in both infected cells and uninfected cells on the same transwell insert. Since it is unlikely that the effect of jasplakinolide will discriminate between RSV-infected cells and uninfected cells, we think the data on the G/F-actin ratio in Figure 1B won't be impacted by this additional information about the RSV infection ratio.

[NOTE: We have removed a figure which was provided for the referees in confidence.]

3. Jasplakinolide is an inducer of actin polymerization but not an inhibitor of actin depolymerization. Indeed, reference 51 (Bobb M.R. et al, 1994, JBC 269) suggests that "Actin filament made by the addition of jasplakinolide were resistant to depolymerization". Therefore, experimental results with jasplakinolide (Figures 1-3) need to revisit with a correct F-actin depolymerization inhibitor.

Response: Thank you for bringing this to our attention. We acknowledge that we should refer to jasplakinolide as an actin-specific reagent that promotes actin polymerization. However, as the reviewer pointed out in reference 51, jasplakinolide has been widely used to stabilize actin filaments and prevent depolymerization, which will still fulfill our goal of ours to mitigate the F-

© 2022. Published by The Company of Biologists under the terms of the Creative Commons Attribution License (https://creativecommons.org/licenses/by/4.0/).

actin depolymerization caused by RSV. We have corrected this in our manuscript accordingly by changing the description of jasplakinolide as an inducer of actin polymerization from the inhibitor of actin depolymerization (please see pages 8-9).

4. Figure 2, Without viral infection rate (and detection of infected cells) it is difficult to understand the results. For example, RSV is known to form syncytia in vitro, but no syncytia formation in vivo or more appropriate in vitro model, e. g., ALI culture. It needs to be determined whether syncytia formation disrupted cell-cell junction. RSV may cause syncytia formation in the 16HBE cell line. Then the syncytia formation is more likely an irreversible event. It must be determined whether the F-actin depolymerization inhibitor can restore RSV-induced CPE, increasing barrier impermeability.

Response: Thank you for making such an excellent point. Please refer to our response to comment 1 regarding the question about the infection rate in RSV-infected 16HBE cells. As for the reviewer's concern about RSV-induced syncytia formation, we evaluated the dysfunction and the structural disruption of the epithelial barrier 24 hours after infection, while syncytia formation was observed by us and other groups to start later than our endpoint. For example, we observed that RSV-induced syncytia are apparent around 48 hours after infection at an MOI of 0.5 (unpublished data), others reported that at an MOI of 1.0, RSV did not induce a high percentage of syncytia formation in A549 cells 24 hours post- infection while such percentage was obvious 48 hours and 72 hours after infection (Mehedi M. et al., PLoS Pathog, 2016). In another study, it was reported that in the first 24 hours following RSV infection, no obvious syncytia were detectable at MOI of 1.0, but by 48 hours after infection, multinucleate syncytia were readily observed in the HEK cultures (Tian J. et al., J Gen Virol, 2013). Whether stabilizing F-actin would affect syncytia formation caused by RSV is a fascinating direction to follow up, but is currently out of the scope of this manuscript.

5. RSV infection causes cytopathic effect (CPE) on an infected cell monolayer. In Fig 4A&B, the observed less cortactin could be the result of the RSV-induced cell death. Besides, UV-RSV is not an appropriate control. Therefore, RSV-induced cortactin reduction should be established at a single-cell level.

Response: Thank you for this insightful suggestion. As for the cytopathic effect of RSV, our lab has characterized that with the same infection condition used in this study, RSV would not induce cell cytotoxicity or apoptosis in 16HBE cells (Rezaee F et al., J Virol, 2013).

Regarding the reviewer's question about cortactin reduction at a single-cell level, we quantified the mean fluorescence intensity of cortactin immunostaining. We found that, in 16HBE cells, the average intensity of cortactin decreased by 58.1% in RSV-infected cells and by 59.0% in uninfected cells (A, n = 50 cells from 10 images). In addition, similar results were also observed in NHBE cells cultured under ALI conditions: the mean intensity of cortactin was reduced by 48.8% in infected cells, and by 40.6% in uninfected cells (B, n = 40 cells from 4 images). Our data suggest that RSV infection reduces cortactin in both infected cells and uninfected cells. As elaborated under comment 1, the observed effects of RSV infection in our model are likely combined results of the direct effect of viral infection and released inflammatory cytokines and chemokines in response to viral infection.

[NOTE: We have removed a figure which was provided for the referees in confidence.]

6. Figure 4E shows that the RSV-infection rate or RSV infection needs to be established in the ALI. Additionally, RSV-induced F-actin depolymerization hasn't been established in the ALI.

Response: Thank you for your feedback. Via a similar approach in our response to comment 1, we imaged the RFP signals as an indicator of RSV infection and analyzed the percentage of RFP-positive NHBE cells 96 hours after infection of RSV at MOI of 2 (A, scale bar, 30 μm). Statistical results showed that the RSV infection ratio in NHBE cells was about 61% (n = 8 images, each image contains approximately 30 cells).

[NOTE: We have removed a figure which was provided for the referees in confidence.]

© 2022. Published by The Company of Biologists under the terms of the Creative Commons Attribution License (https://creativecommons.org/licenses/by/4.0/).

To characterize RSV-induced F-actin depolymerization in NHBE cells cultured under ALI conditions, we determined the mean fluorescence intensity of F-actin in RSV-infected cells and RSV-uninfected cells. The average intensity of F-actin reduced significantly both in RSV-infected cells (by 69.7%)and uninfected cells (by 64.9%) compared with control cells; moreover, the decrease in F-actin intensity was comparable between infected cells and uninfected cells (n = 40 cells from 4 images).

[NOTE: We have removed a figure which was provided for the referees in confidence.]

7. Figure 4 F-H, the results are difficult to interpret without RSV detection in the infected epithelium.

Response: We recognize that it is essential to show evidence of RSV infection in vivo. In our experience, RSV infection usually does not cause a massive infection in vivo that can be reliably detected by immunohistochemistry staining. However, our previous publications have established proof of RSV infection in the same mouse model used in this study. For example, quantitative real-time PCR analysis revealed an increased mRNA expression of a specific viral N gene in lung homogenates of RSV-infected mice on day 4 post-inoculation. In contrast, no mRNA expression of the viral N gene was detected in control or UV-RSV inoculated mice. Furthermore, the plaque-forming analysis demonstrated visible plaques in HEp-2 cells inoculated with diluted lung lysate from RSV-infected mice. Of note, we used UV- inactivated RSV as a control, lung homogenates from control groups yielded no plaques, and the UV- RSV group produced a negligible number of small plaques, indicating a lack of efficient viral replication (Smallcombe CC et al., Am J Physiol Lung Cell Mol Physiol, 2019). Taken together, we are confident that RSV infection is occurring in vivo in our animal model.

8. Figure 5-6, Cortactin involvement needs to be established in the RSV infection before further characterization of cortactin in cells

Response: Thank you for stressing this issue again. We addressed the concerns about RSV-induced reduction of cortactin in our response to comment 5.

Minor comments:

1. Fig 4E, Orthogonal (XZ or YZ) view will help to observe the respiratory epithelium (Here, only XY plane was presented). Moreover, it is not specified which Z-plane was taken for this image preparation. The result is not convincing without displaying RSV infection in the apical surface. Again, RSV-infected cells are not detected here.

Response: Thank you for this detailed observation. We did not take z-stack images while collecting images in Fig 4E, and the Z-plane we took was the apical surface based on the patterns of TJ/AJ protein and the outline of cells during confocal imaging, and we have now included such information in Materials and Methods session (page 20, line 517-518). For the representative image of RSV infection and statistical results in NHBE cells, please refer to our response to comment 6.

2. According to reference 11, the authors measured TEER just 7-day post-seeding in ALI culture but did not show what the epithelium looked like during that time. Based on the TEER measurements of previous reports, it can assume that the epithelial cell barrier will not be functional enough in ALI culture during 7-day post-seeding.

Response: The timing of experiments 7 days post-seeding was for 16HBE cells cultured in a liquid-liquid environment, not for an air-liquid environment. 16HBE cells with a TEER > 500 $\Omega \times cm^2$ were selected for further experiment to evaluate barrier function. We now included culture time for TEER measurements in the Materials and Methods session to avoid confusing our readers (page 18, line 454). For details of ALI culture, please see our response to minor comment 4.

3. According to reference 11 (also used in Reference 10 and 30), air-liquid interface (ALI) culture was maintained for only 14 days. Multiple groups (Michi et al., 2021, Broadbent et al., 2020, and Osan et al., 2021) suggested ALI culture needs differentiation at least 3 weeks; however, 4 weeks is preferable. The higher differentiation tends to mimic the biophysical properties of in vivo airways.

© 2022. Published by The Company of Biologists under the terms of the Creative Commons Attribution License (https://creativecommons.org/licenses/by/4.0/).

Response: We did cite several references for the method regarding the air-liquid interface (ALI) culture of NHBE, but we have included technical details of our experiments in both the Materials and Methods session (page 18, lines 459-464) and the legend for Figure 3 (page 37, lines 948-951). In our study, ALI cultures were maintained 4-for 5 weeks (27-35 days) with a TEER > 1500 Ω × cm^2 before infections and experiments, according to the reviewer, this should be appropriately differentiated and able to mimic in vivo properties.

Reviewer 3 Advance Summary and Potential Significance to Field...

The authors of this study demonstrate convincingly that infections of (airway) epithelial cells with Respiratory syncytial virus(RSV) reduces the expression of cortactin followed by accompanied downstream effects previsouly established by others in different systems, such as compromized Rap/Rac signaling and epithelial junction destabilization. The authors also show that their airway epithelial cell model displays phenotypes comparable to RSV infections if cortactin expression is eliminated by CRISPR/Cas9. The reduction of cortactin expression effected by RSV infection requires replicative activity of the virus, but the mechanistic connection between viral replication and downregulation of cortactin expression remains unknown. This is a quite complete study that complements previous knowledge both on RSV biology and cellular as well as organismic cortactin functions. The only major experiment missing in my view was to perform RSV infections on the cortactin-depleted 16HBE cells shown and characterized in Fig. 5. Given that RSV can still enter the cells (which I assume but would have to be clearly demonstrated), this experiment would allow the authors to judge or estimate at least the relative contribution that the downregulation of cortactin is playing alongside with potential additional changes caused by RSV infection. If the observed downregulation of cortactin expression by RSV has a significant contribution to the phenotypes developed upon RSV infections (which is assumed at present and the main conclusion of the study), the extent of additional effects caused by RSV in the absence of cortactin should be eliminated or at least reduced. This applies in particular to the reduction of TEER and increase of dextran flux already observed in the cortactin null cell line (Figure 5), but also, of course, to the partial disintegration of epithelial junctions, as judged by ZO-1, E-cadherin, occluding or beta-catenin staining. In conclusion, in case this experiment is provided and displays a clear outcome (whatever this may be), I would strongly vote for publication of the study in due time. Concerning the Figures, in particular in the context of potential addition of data. The figures are nice and well understandable, even without much reading of the legends (I much like the model in Figure 9 – this is beautiful), but if additional space is required, some of them could certainly be combined or moved to the Supplement (with a specific suggestion given below).

Reviewer 3 Comments for the Author...

There are only a few important points from my side at this stage that read as follows:
1) I am not sure how relevant Figure 3 is as stand alone really. This could be combined with the previous Figure or moved to the supplement.

Response: Thank you for this suggestion, we have combined Figure 3 with Figure 2 in our updated manuscript (now Figure 2 in the updated manuscript).

2) The headline of Fig. 5 reads…"Epithelial barrier integrity is disrupted in cortactin knockout…". Looking at the data, "disrupted" seems a bit strong, as I feel that the effect at best constitutes a partial weakening and "thickening" of junctional linings between cells (this is at least how it appears in most cells at the currently shown magnification). Given previous reports in the literature, I assume the apparent thickening constitutes a more disorganized, zig-zag arrangement of the junctional connections between cells, but no complete disruption. This could be a bit confusing to readers, so in general, the images shown concerning junctional integrity in different treat6ments in the paper (RSV infections and cortactin-KO) could be complemented by higher mag images clearly defining what's going on. This would probably also work if selecting just one condition, like the cortactin KO, which will likely give the cleanest and clearest phenotype in this respect, as representative example – but this will of course be up to the authors.

© 2022. Published by The Company of Biologists under the terms of the Creative Commons Attribution License (https://creativecommons.org/licenses/by/4.0/).

Response: Thank you for this excellent observation and advice. We now included images of higher magnification for the immunostaining of TJ/AJ proteins in WT and cortactin KO cells in Figure 4F to clarify the phenotype of the barrier disruption. We have included this additional data in our manuscript (page 10, lines 227-230).

3) Concerning the characterization of CRISPR-Cas9- mediated cortactin gene disruption. This is nice, and I understand the authors to have generated individual KO clones. However, it remained unclear to me when briefly glancing through the Methods section whether the authors are showing data for just one clone in Figure 5 or a mixture of clones perhaps, and whether they can recapitulate phenotypes in several, optimally up to 3-5 clones, which would really exclude non-specific, off-target effects in my view. Another possibility of excluding off-target effects (which I do not find likely here personally though) could be to rescue the phenotypes with GFP-cortactin used in the experiment in Figure 6, but this might be asking for a bit much because possibly technically challenging. So instead, I would recommend providing a Supplementary Figure with results from a few independently generated cortactin null clones, which could very well complement or replace the need for a rescue. However, I have also just noticed that the authors have refrained from providing any information on how the cortactin null clones were characterized at the genomic level. I would strongly recommend performing TIDE sequencing (or alike, see e.g. PMID: 25300484) of a few CRISPR-clones selected based on Western data, allowing to examine the precise mutations introduced and their frequency of occurrence, as well as potentially excluding the presence of any wildtype alleles in individual clones.

Response: Thank you for these great suggestions. The data we showed in Figure 5 in the previous manuscript was from one clone, however, we did repeat the experiments with 2 more independently generated cortactin KO clones and observed similar results. We agree with the reviewer it is critical to exclude non-specific, off-target effects in our experiments. Therefore, we updated the TEER measurements and dextran efflux assay data in Figures 4C and 4D to include pooled data from 3 independent KO clones. As for the immunofluorescent staining towards TJ and AJ proteins, representative images from one clone are shown in Figures 4E and 4F, while images from 2 additional clones are now provided in Supplementary Figures 1C and 1D. We also added images of the RSV virus, which expresses RFP upon replication, in WT and cortactin KO cells after infection as Supplementary Figure 1B as the reviewer suggested. Our data showed that the RSV infection was comparable between WT and KO 16HBE cells. The manuscript has been updated accordingly.

As for how the cortactin null clones were characterized at the genomic level, we performed PCR towards the genomic DNA isolated from three KO clones used in the current study. Then the PCR products were sequenced by Sanger sequencing and analyzed by the Inference of CRISPR Edits analysis (ICE analysis, bioRxiv 251082; doi: https://doi.org/10.1101/251082) from Synthego (Menlo Park, CA). All KO clones showed indels with high frequencies of occurrence (□ 99%). This new evidence indicated successful genomic editing and is consistent with our Western blot results which were used to select and validate KO clones. We have included the representative results of CRISPR editing as Supplementary Figure 1A and updated our manuscript to provide more details, especially in the Materials and Methods session (pages 22-23).

4) I did not understand why Figure 6A shows an admittedly less abundant but still clearly detectable band at the molecular size of GFP in the GFP-cortactin sample on the right, although solely transfected with GFP-cortactin I guess. This is probably not uncommon though, but likely suggests degradation of ectopic cortactin down to the size GFP. This should not be left uncommented I think, and I would also encourage showing entire blots (at least in the Supplement) so that the reader can appreciate the extent of GFP-cortactin degradation in this experiment. Indeed, the extent of improvement of TEER in GFP-cortactin-transfected, RSV-infected cells was statistically significant, yet quite limited (as compared to the extent of decrease of FITC-dextran flux, for instance), so this fact might be explained, at least in part, by quite strong GFP-cortactin degradation. Whatever the case, additional information in this context might give the reader the chance at least to judge themselves how all these data fit together.

Response: Thank you for these excellent insights. We have included the full-length blot for GFP in Figure 5A as Supplementary Figure 2E to provide additional information. The lysates of GFP-cortactin overexpressing cells displayed additional bands at lower molecular weights, which could

© 2022. Published by The Company of Biologists under the terms of the Creative Commons Attribution License (https://creativecommons.org/licenses/by/4.0/).

be a result of the GFP-cortactin degradation after RSV infection. We have described and discussed the bands at lower molecular weights on the blot and the extent of GFP-cortactin degradation on page 10, lines 245-247 in the updated manuscript.

Regarding the limited improvement of TEER by overexpressing GFP-cortactin and the discrepancy between the rescues effect of GFP-cortactin on TEER and dextran flux, we'd like to provide more information on the differences between these two parameters. The electrical resistance and the permeation of macromolecule tracers such as dextran (we used 4 kD dextran in this study) are both indicators of paracellular permeability, electrical resistance is a measurement of the transjunctional flow of small inorganic molecules (mostly ions), while the dextran permeability characterizes the flux of high molecular weight biomolecules across the cellular barrier (reviewed in detail by Radoslaw Bednarek, Methods Protoc., 2022). Therefore, results from these assays are not linearly correlated and their degree of correlation worsens as the molecular size of the solute increases (Madara, J.L. & Dharmsathaphorn, K. J. Cell Biol. 1985). In our study, the reflection of restored barrier integrity by the decrease in FITC- dextran flux was more noticeable than the improvement in TEER, probably because of the size differences between the molecules they measured. We have updated our manuscript to include this additional information in the Discussion (page 16, lines 395-402).

5) As already elaborated on in the general comments above, the most significant experiment in my view concerning this story would be to take a well-characterized, cortactin-deficient clone and explore the extent of changes still induced by RSV infections!

Response: Thank you for this great feedback. We examined the barrier permeability as well as the structure in RSV-infected cortactin KO cells as advised. It is worth mentioning that three independent clones were used to exclude non-specificity from the knockout process. Through measurements of TEER, dextran efflux assay, and immunostaining towards the apical junctional complex, we observed that RSV infection caused insignificant additional effects in KO cells, these data imply that cortactin is a major downstream factor mediating RSV-induced barrier disruption. This evidence is now presented in Figure 4C-F and described on page 10 lines 232-235.

We also assessed the activity of Rap1 in RSV-infected KO cells, and we did not find a significant additive decrease in Rap1 activity compared to KO cells (Figure 6C, 6D). We have included and explained these data on pages 11-12 lines 275-280 in our updated manuscript.

Second decision letter

MS ID#: JOCES/2022/259871

MS TITLE: Respiratory syncytial virus disrupts airway epithelial barrier by decreasing cortactin expression and destabilizing F-actin

AUTHORS: Nannan Gao, Andjela Raduka, and Fariba Rezaee
ARTICLE TYPE: Research Article

We have now reached a decision on the above manuscript.

To see the reviewers' reports and a copy of this decision letter, please go to: https://submit-jcs.biologists.organd click on the 'Manuscripts with Decisions' queue in the Author Area. (Corresponding author only has access to reviews.)

As you will see, two reviewers gave favourable reports and support publication. In contrast, reviewer 2 still thinks the paper has major issues that preclude publication. I think the issues they have raised concerning RSV detection and level of infection are a concern and need to be addressed. Basically, you need to provide images that demonstrate the levels of infection with the MOI you are using in your assays. I hope that you will be able to carry these out because I would

© 2022. Published by The Company of Biologists under the terms of the Creative Commons Attribution License (https://creativecommons.org/licenses/by/4.0/).

like to be able to accept your paper, depending on further comments from reviewers if we feel it is still necessary.

Please ensure that you clearly highlight all changes made in the revised manuscript. Please avoid using 'Tracked changes' in Word files as these are lost in PDF conversion.

I should be grateful if you would also provide a point-by-point response detailing how you have dealt with the points raised by the reviewers in the 'Response to Reviewers' box. Please attend to all of the reviewers' comments. If you do not agree with any of their criticisms or suggestions please explain clearly why this is so.

Reviewer 1

Advance summary and potential significance to field

My concerns have been addressed satisfactorily. I have no further comments.

Comments for the author

My concerns have been addressed satisfactorily. I have no further comments.

Reviewer 2

Advance summary and potential significance to field

Overall, the RSV infection has not been optimized in this in vitro model. Thus, the results are not convincing at all.

Comments for the author

Comments to the authors' response on #1: The authors have failed to provide convincing evidence of infected cells in the representative image (Fig1A or Fig S1B). Because: first, at 24 hr post-infection at an MOI = 0.5 63% of RSV-infected cells is doubtful in any in vitro model. This reviewer thinks 63% of RSV-infected cells at 24-hour post-infection in the transwell is virtually impossible. Thus, the detection strategy was not optimal or did not provide an accurate measurement. It can be speculated that the observed red dots were not necessarily RFP in the RSV-RFP infected cells. Different lab research data with fluorescence viruses, e.g., RSV-GFP, could see those background signals. Second, RFP in the RSV-RFP infected cells is expected to be diffusive, not punctuated, because the fluorescence protein expression is generally diffusive and distributed throughout the cell. The author's cited paper (Guerrero-Plata et al., 2006) has shown RFP expression thought out of the RSV-RFP infected cells. Thus, the punctated spot can be a background signal, not the RFP. Third, it was also confusing whether they used 16 HBE confluent monolayer or 16HBE differentiated monolayer (epithelium characterization is required not limited to TEER value).
Comments to the authors' response on #2: As RSV-infected cells detection has not been optimized (see comment 1), the determination of RSV-induced F-actin depolymerization in the infected cells might not have been accurate. For example, they found that RSV-infected cells showed 70% F-actin intensity reduction. The 70% F-actin reduction may cause a substantial cell integrity loss. Therefore, RSV infection may cause cell damage 24 hr post-infection. With a 63% infection rate, the cell layer can't be expected in the RSV-infected Truswell or monolayer.
Comments to the authors' response on #3: As RSV-infected cells detection has not been optimized (see comment 1), no conclusion can be drawn.

Comments to the authors' response on #4: The authors' explanation about jasplakinolide is incorrect because Bobb M.R. et al., 1994, JBC 269 suggested it is an actin polymerization inducer. Furthermore, the paper indicated that jasplakinolide-induced actin is resistant to depolymerization, different from the authors' response. Thus, to establish the results, the authors need to use the correct actin depolymerization agent (Figs 1-3).

© 2022. Published by The Company of Biologists under the terms of the Creative Commons Attribution License (https://creativecommons.org/licenses/by/4.0/).

Comments to the authors’ response on #5: Fig 2 issue can quickly be addressed by detecting RSV-infected cells. However, as RSV-infected cells detection has not been optimized (see comment 1), it is difficult to assess the result.
Comments to the authors’ response on #6: RSV infection reduced cortactin regardless of infected (48.8%) or uninfected (40.6%) at a similar rate, which is quite unbelievable. When the infection rate is 63% at 24 hour-post infections, with 50% cortactin reduction in the whole epithelium (transwell), what is the expected rate of cortactin reduction at 48-hour post-infection? The authors also suggested that RSV does not induce cell cytotoxicity or cell death mechanism.

Reviewer 3

Advance summary and potential significance to field

I think the authors have done a great job in trying to address my previous comments and concerns, so I am satisfied with all the changes made. This is an interesting manuscript that should be published rapidly.

Comments for the author

Very minor point:
There is one strange wording (already there in the abstract of the previous version that I had missed), which reads (lines 49/50):
"The activity of Rap1, on donwnstream...?...of cortactin, declined after..."
I guess the authors meant to say something like: one downstream target/"effector"...something like this (effector probably not being the best choice though because reserved to a certain extent in the field for downstream targets of small GTPases I assume...)

Second revision

Author response to reviewers' comments

Response to the Reviewers:

Reviewer 1 Advance Summary and Potential Significance to Field:
My concerns have been addressed satisfactorily. I have no further comments.

Reviewer 1 Comments for the Author:
My concerns have been addressed satisfactorily. I have no further comments.

Response: We thank the reviewer for previous comments and suggestions which played an important part in improving our manuscript. We are glad to learn that our response is satisfactory to the reviewer.

Reviewer 2 Advance Summary and Potential Significance to Field:

Overall, the RSV infection has not been optimized in this in vitro model. Thus, the results are not convincing at all.

Reviewer 2 Comments for the Author:

Comments to the authors’ response on #1: The authors have failed to provide convincing evidence of infected cells in the representative image (Fig1A or Fig S1B). Because: first, at 24 hr post-infection at an MOI = 0.5, 63% of RSV-infected cells is doubtful in any in vitro model. This reviewer thinks 63% of RSV-infected cells at 24-hour post-infection in the transwell is virtually impossible. Thus, the detection strategy was not optimal or did not provide an accurate measurement. It can be speculated that the observed red dots were not necessarily RFP in the RSV-RFP infected cells.

© 2022. Published by The Company of Biologists under the terms of the Creative Commons Attribution License (https://creativecommons.org/licenses/by/4.0/).

Different lab research data with fluorescence viruses, e.g., RSV-GFP, could see those background signals. Second, RFP in the RSV-RFP infected cells is expected to be diffusive, not punctuated, because the fluorescence protein expression is generally diffusive and distributed throughout the cell. The author's cited paper (Guerrero-Plata et al., 2006) has shown RFP expression thought out of the RSV-RFP infected cells. Thus, the punctated spot can be a background signal, not the RFP. Third, it was also confusing whether they used 16 HBE confluent monolayer or 16HBE differentiated monolayer (epithelium characterization is required not limited to TEER value).
Response: We thank the reviewer for these comments.

1. To address the reviewer's concerns about the detection and the level of RSV infection, we performed immunostaining using antibodies against RSV G Glycoprotein (RSV-G) and conducted confocal imaging and analysis in addition to our previous response (A-B). 16HBE monolayers were also stained with fluorescein-conjugated Phalloidin and DAPI. Panel C shows representative images for control cells or RSV-infected cells 24 hours after infection at an MOI of 0.5 (Scale bar, 30 μm). We also included a 'No Primary Ab' group to examine if there is any non-specific signal from the RSV-G antibody. Comparing the images of RSV-infected cells with control cells and the 'No Primary Ab' group, it is obvious that the green signals in panel C are specific. The statistical result indicates that about 63% of the 16HBE cells showed positive staining of RSV-G (D, n = 10 images, 56-95 cells per image), which is similar to the ratio of RSV-RFP-positive cells (63.05¤3.141%, A-B). Others have reported the percentage of RSV infection in A549 cells, a lung epithelial cell line at 24h post-infection at different MOIs (Naseem Ahmed Khan et al., mSphere, 2020, PMID: 32461278). They found that the proportion of RSV-infected cells was 40% at an MOI of 0.3, 70% at an MOI of 1, and 95% at an MOI of 3, indicating that the 63% infection ratio we concluded using two different visualization approaches is within a reasonable range.

[NOTE: We have removed a figure which was provided for the referees in confidence.]

2. As for the concerns about the puncta pattern of RSV-RFP we observed, it's worth mentioning that the diffusive RFP signals in our cited paper (Figure 1, Guerrero-Plata et al., Am J Respir Cell Mol Biol, 2006) represented rrRSV-infected cells observed under fluorescent light, not confocal images of immunostaining experiment on fixed cells. In our experience, we have observed that RFP expression is distributed throughout the infected cells on transwell membranes under the fluorescent microscope. However, qualifying the infection ratio with such images is not optimal because of the challenging nature of imaging the transwell membranes suspended in the media. In our previous response and manuscript, 16HBE cells were fixed by 4% PFA, permeabilized by 0.1% Triton X-100 and subjected to direct imaging against rrRSV- RFP. The red 'dots' were not detected in control cells from the same experiment and visualized under the same imaging setting, indicating that they are not non-specific signals. One possible explanation is that the staining procedure we used compromised the detection of the diffused RFP, which is not an uncommon phenomenon when visualizing fluorescent proteins on PFA fixed samples.
3. For the last comment, we have described that we used confluent monolayers of 16HBE cells in our manuscript.

Comments to the authors' response on #2: As RSV-infected cells detection has not been optimized (see comment 1), the determination of RSV-induced F-actin depolymerization in the infected cells might not have been accurate. For example, they found that RSV-infected cells showed 70% F-actin intensity reduction. The 70% F-actin reduction may cause a substantial cell integrity loss. Therefore, RSV infection may cause cell damage 24 hr post-infection. With a 63% infection rate, the cell layer can't be expected in the RSV-infected Truswell or monolayer.
Response: We thank the reviewer for this comment. Regarding the concerns about the detection of RSV-infected cells, please see our response to comment 1. As for the comments on the F-actin intensity reduction, in our previous response, we showed the average intensity of F-actin declined in RSV-infected cells to 70.2% of control cells, not by 70.2%. Additionally, we did not observe a severe loss of cells in the monolayer 24h after infection, as evidenced by the overall monolayer outlines when performing the immunofluorescence staining of F-actin and DAPI (Figure 1A and the image under comment 1 in this response). Our lab has also characterized that, with the same condition, RSV does not induce cell cytotoxicity in 16HBE cells (Rezaee F. et al., J Virol, 2013).

© 2022. Published by The Company of Biologists under the terms of the Creative Commons Attribution License (https://creativecommons.org/licenses/by/4.0/).

Comments to the authors' response on #3: As RSV-infected cells detection has not been optimized (see comment 1), no conclusion can be drawn.
Response: We thank the reviewer for the comment, reviewer's concern about the detection of RSV infection was addressed in our response to comment 1.

Comments to the authors' response on #4: The authors' explanation about jasplakinolide is incorrect because Bobb M.R. et al., 1994, JBC 269 suggested it is an actin polymerization inducer. Furthermore, the paper indicated that jasplakinolide-induced actin is resistant to depolymerization, different from the authors' response. Thus, to establish the results, the authors need to use the correct actin depolymerization agent (Figs 1-3).
Response: We thank the reviewer for bringing this to our attention. As an actin-binding compound, jasplakinolide is commonly used as an actin stabilizer, as well as an inducer of actin polymerization. Jasplakinolide is a cyclic depsipeptide that contains structural features shared among actin stabilizing substances (Allingham, J.S. et al., Cell Mol Life Sci,2006), and has been used in various studies to stabilize actin filament (Posey S.C. and Bierer B.E., J Biol Chem,1999; Visegrády B. et al., FEBS letters, 2005; Zhang X. et al., PLoS ONE, 2012). We observed increased depolymerization after RSV infection in our study, which prompted us to use jasplakinolide to stabilize actin network and examine its effect on barrier integrity and function after RSV infection. We also validated the ability of jasplakinolide to stabilize actin network in our assays in Figures 1B and 1C, evidenced by the reversion of increased G/F-actin ratio in RSV-infected cells after jasplakinolide treatment, We recognized that, other than the citation on its original discovery, reports on jasplakinolide as an actin stabilizer are missing in our manuscript, so now we have included these references in our updated manuscript (page 8, line 168). We also acknowledge that our description of "jasplakinolide has been widely used to … prevent depolymerization" in our previous response was inaccurate and did not effectively convey our rationale for using this compound, we have clarified jasplakinolide as an actin stabilizer in our manuscript (page 8, lines 167-168) with the edited content in red.

Comments to the authors' response on #5: Fig 2 issue can quickly be addressed by detecting RSV-infected cells. However, as RSV-infected cells detection has not been optimized (see comment 1), it is difficult to assess the result.
Response: We thank the reviewer for stressing this issue again. Please refer to our response to comment 1 regarding the detection of RSV-infected cells.

Comments to the authors' response on #6: RSV infection reduced cortactin regardless of infected (48.8%) or uninfected (40.6%) at a similar rate, which is quite unbelievable. When the infection rate is 63% at 24 hour- post infections, with 50% cortactin reduction in the whole epithelium (transwell), what is the expected rate of cortactin reduction at 48-hour post-infection? The authors also suggested that RSV does not induce cell cytotoxicity or cell death mechanism.
Response: We thank the reviewer for the comments. Regarding our observation that RSV infection reduces cortactin in both infected cells and uninfected cells on the same transwell insert, one possible explanation is that the observed effects of RSV infection on cortactin in our model reflect both a direct effect of viral infection and a consequence of released inflammatory cytokines and chemokines in response to viral infection. It has been well documented that RSV triggers the expression and secretion of cytokines and chemokines from airway epithelial cells (Noah T. L. and Becker S., Am J Physiol Lung Cell Mol Physiol, 1993; Zhang Y. et al., J Virol, 2001; Smallcombe C.C. et al., Am J Physiol Lung Cell Mol Physiol, 2020), and the resulted release of cytokines such as IL-4, IL-13, and IFNγ has been shown to disrupt epithelial barrier integrity (Bruewer M. et al., FASEB J, 2005; Utech M. et al., Mol Biol Cell, 2005; Saatian B. et al., Tissue Barriers, 2013). The mechanism and pathways we revealed in this study could also mediate such indirect impacts of RSV and would be an interesting direction to pursue in the future. As for the change in cortactin at 48h post-infection, we included Western blot data in Figures 3A and 3C in our original submission, which showed a 37.8% decrease in the protein level of cortactin, we also observed that there was no noticeable decrease from 24h to 48h post-infection. We acknowledge that the extent of decrease is slightly different between immunostaining and western blot, which could be attributed to discrepancies between these different detection methodologies. As for the cytopathic effect of RSV specific to the experimental conditions we used in our manuscript, our lab has characterized that, with the same condition, RSV does not induce cell cytotoxicity or apoptosis in 16HBE cells (Rezaee F. et al., J Virol, 2013). Similar results were also reported by other groups on RSV infection of the A549 cell line (Naseem Ahmed Khan et al., mSphere, 2020).

© 2022. Published by The Company of Biologists under the terms of the Creative Commons Attribution License (https://creativecommons.org/licenses/by/4.0/).

Reviewer 3 Advance Summary and Potential Significance to Field:
I think the authors have done a great job in trying to address my previous comments and concerns, so I am satisfied with all the changes made. This is an interesting manuscript that should be published rapidly.
Response: We thank the reviewer for providing constructive comments and concerns which helped to improve our manuscript significantly. We are glad to hear that the reviewer found our response adequate and satisfactory.

Reviewer 3 Comments for the Author:
Very minor point:

There is one strange wording (already there in the abstract of the previous version that I had missed), which reads (lines 49/50):

"The activity of Rap1, on donwnstream...?...of cortactin, declined after..."

I guess the authors meant to say something like: one downstream target/"effector"...something like this (effector probably not being the best choice though because reserved to a certain extent in the field for downstream targets of small GTPases I assume...)

Response: We thank the reviewer for pointing this out. The sentence now reads “The activity of Rap1, one downstream target of cortactin, declined after RSV infection…” (page 3, line 49)

Third decision letter

MS ID#: JOCES/2022/259871

MS TITLE: Respiratory syncytial virus disrupts airway epithelial barrier by decreasing cortactin and destabilizing F-actin

AUTHORS: Nannan Gao, Andjela Raduka, and Fariba Rezaee
ARTICLE TYPE: Research Article

I am happy to tell you that your manuscript has been accepted for publication in Journal of Cell Science, pending standard ethics checks.

© 2022. Published by The Company of Biologists under the terms of the Creative Commons Attribution License (https://creativecommons.org/licenses/by/4.0/).
